# Inhibition of angiogenesis in endothelial cells by Human Lysyl oxidase propeptide

**DOI:** 10.1038/s41598-018-28745-8

**Published:** 2018-07-11

**Authors:** Ragavachetty Nagaraj Nareshkumar, Konerirajapuram Natarajan Sulochana, Karunakaran Coral

**Affiliations:** 1 R.S. Mehta Jain Department of Biochemistry and Cell Biology, Vision Research Foundation, 41, College road, Chennai, India; 2 School of Chemical and Biotechnology, SASTRA University, Thanjavur, India

## Abstract

Angiogenesis is a critical process involved in normal physiology. Pathological angiogenesis is observed in vascular diseases and neoplasia. The propeptide domain of LOX (LOX-PP) has been shown to inhibit tumorigenesis in various cancers. In this study, we explored the role of both overexpressed and recombinant LOX-PP in naïve human umbilical vein endothelial cell with the addition of vascular endothelial growth factor (VEGF). Primarily, we observed a significant reduction in the angiogenesis signaling pathways upon LOX-PP overexpression by proteomic analysis. Further functional analysis showed that the VEGF induced cell proliferation, migration, adhesion and tube formation was inhibited by LOX-PP. Moreover, LOX-PP arrested cells at S-phase, reduced F-actin levels and decreased phosphorylation of focal adhesion kinase (FAK) and extracellular signal regulated kinase (ERK). The anti-angiogenic effect of LOX-PP was further confirmed by the reduction in the vascular network formation in chick chorioallantoic membrane (CAM). These results indicate that inhibition of angiogenesis events is not only achieved by overexpressing LOX-PP but also by addition of rLOX-PP. Taken together our findings discovered the anti-angiogenic role of LOX-PP in endothelial cells which suggests that harnessing this potential can be a promising strategy to inhibit angiogenesis.

## Introduction

Angiogenesis is a dynamic process that involves cell proliferation, migration, adhesion and tube formation in endothelial cells orchestrated by proangiogenic mediators and anti angiogenic factors^1^. This process is tightly balanced by many growth factors, endogenous molecules and intracellular signaling pathways^2^. A shift in this balance leads to pathological uncontrolled angiogenesis as seen in rheumatoid arthritis, psoriasis, proliferative diabetic retinopathy, tumor metastasis etc^2^. There is a growing interest among researchers to target molecules of the pro- and anti-angiogenic pathways as therapeutic modalities.

VEGF being an important pro-angiogenic molecule, is increased in various pathological conditions like proliferative diabetic retinopathy, rheumatoid arthritis^3^, psoriasis^4^ etc. Conventionally, VEGF is controlled by administration of anti-VEGF medications viz Bevacizumab, Ranibizumab, Pegaptanib and Aflibercept. Although anti-VEGF therapy is clinically helpful, some patients show nonresponse and some pose potential systemic side effects that includes proteinuria, hypertension, thromboembolic events like stroke, gastrointestinal perforation, myocardial infarction and ocular complications like vitreous haemorrhage, macular hole, retinal tear and tractional retinal detachment^5^. Consequently, the search for a new, ideal and a potent anti-angiogenic molecule is still underway.

Lysyl oxidase (LOX) (protein-6-oxidase) is an enzyme essential for the biosynthesis of functional extracellular matrices by cross linking collagen and elastin^6,7^. LOX, secreted as a 50 kDa immature precursor, is cleaved extracellularly into a 32 kDa active mature lysyl oxidase enzyme and an 18 kDa lysyl oxidase propeptide (LOX-PP) by the bone morphogenetic protein −1 (BMP-1)^8–11^. The *LOX* gene, also called as the ras recision gene (*rrg*), is reported to inhibit the transforming activity of H-RAS oncogene in NIH 3T3 cells^12,13^. The RAS transforming inhibitory activity of LOX is mapped to the LOX-PP domain^14^, which inhibits the transformation in Her-2/neu driven NF639 breast cancer cells^15^. LOX-PP also inhibits FGF-2 stimulated DNA synthesis and cell growth by impeding the binding of FGF-2 to its receptor in an uncompetitive manner^16^. LOX-PP is reported to prevent PI3K, AKT, ERK and NF-kB activation, thereby inhibiting tumorigenesis in breast, prostate and pancreatic cancers^14,17–19^. It is also proven to interact with the receptor-type protein tyrosine phosphatase kappa (RPTP-κ) and inhibits β-catenin transcriptional activity; thereby negatively regulating the pro-oncogenic β-catenin signaling in lung cancer cells^20^ whereas in breast cancer cells it represses the estrogen receptor alpha transcriptional activity by interacting with ubiquitously expressed transcript (UXT)^21^.

Although there are numerous studies suggesting LOX-PP as an anti-tumorigenic molecule, there is a report alluding that recombinant LOX-PP inhibits TNF-α stimulated aortic smooth muscle cell proliferation and DNA synthesis by down regulating MMP-9 and extracellular signal-regulated kinase (ERK) expression^22^. These observations prompted us to posit the anti-angiogenic function of LOX-PP in endothelial cells. In the current study, we found through proteomics analysis that the major angiogenic signaling pathways were down regulated upon LOX-PP overexpression. Importantly, LOX-PP reduced cell proliferation, migration, adhesion, tube length and the phosphorylation status of pFAK and pERK, thereby repressing angiogenesis in HUVECs. This was further confirmed *in vivo*, by the reduced vascular network formation in chick chorioallantoic membrane (CAM) highlighting the anti-angiogenic potency of LOX-PP.

## Results

### LOX-PP cloning and confirmation

LOX-PP sequence, without the signal peptide was cloned in the pQE 30Xa bacterial expression vector and digested with *Hind III* & *Stu I* restriction enzymes resulted in an insert fragment of 441 bp (Fig. S1a,b). The LOX-PP sequence, with signal peptide was cloned into the pcDNA3.1/His A, a mammalian expression vector and digested with *Hind III* & *Bam HI* restriction enzymes yielded an expected insert of 507 bp (Fig. S1c,d). The identity of these insert was confirmed by DNA sequencing which showed no mutations.

### LOX-PP overexpression and Protein purification

**Figure 1:**
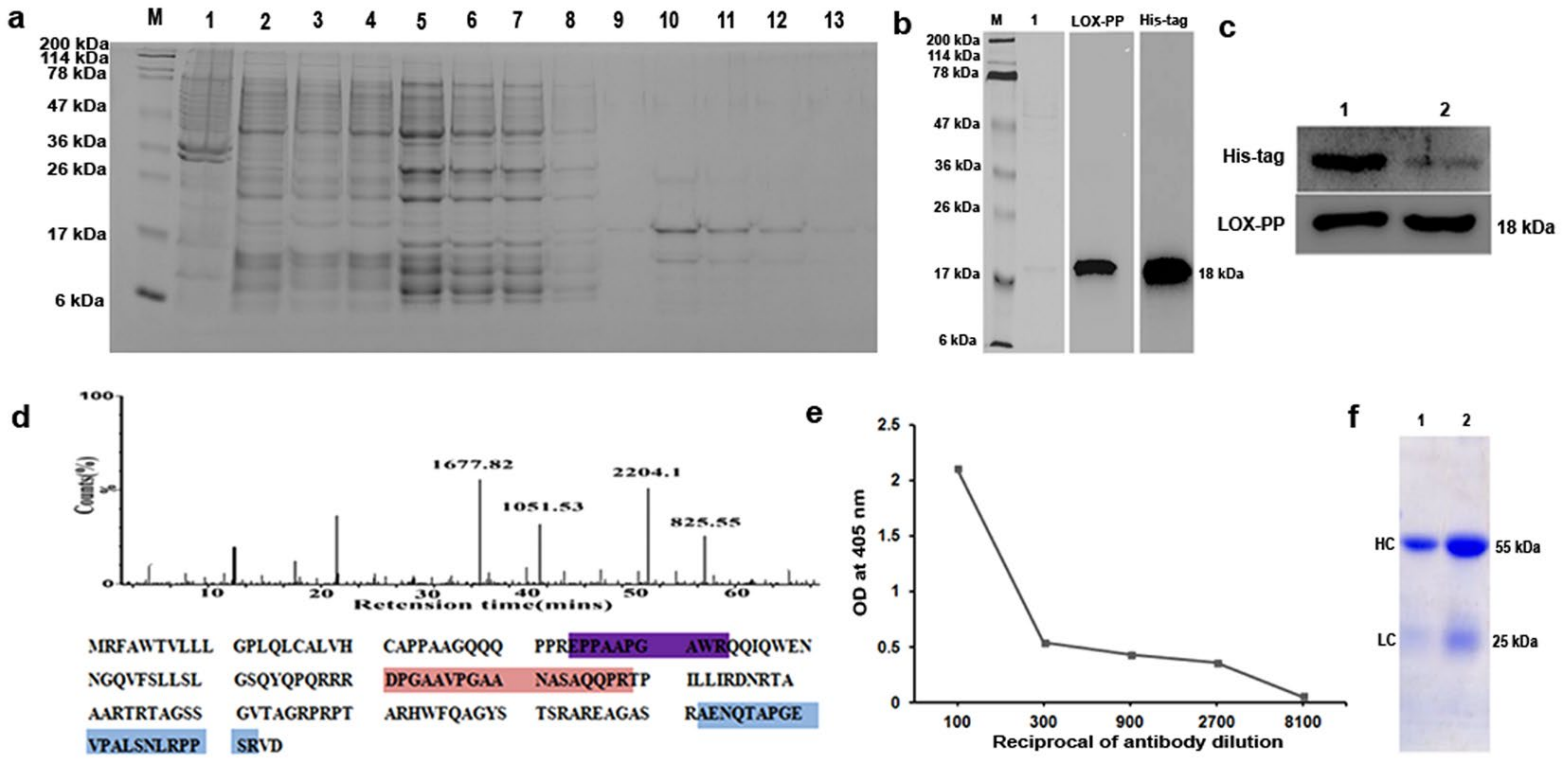
LOX-PP protein purification and antibody production: (**a**) SDS-PAGE of purified LOX-PP using Ni-NTA agarose (Lane-M: Mw Marker, lane-1: Crude, lane-2: Unbound, lane-3 to 8: washes 1 to 6, lane-9 to 13: Elution −1 to 5). (**b**) Western blot for His-tag and LOX-PP in purified protein (M - Mw marker, lane-1: purified protein stained with coomassie stain). The corresponding full length blots are represented in Supplementary Fig. 21. (**c**) Western blot of purified protein post His-tag cleavage using factor Xa protease (Lane-1: His-tag uncleaved, Lane-2: His-tag cleaved. The corresponding full length blots are represented in Supplementary Figs 22 and 23. (**d**) Mass spectrum of the purified LOX-PP and its protein coverage map. (**e**) Direct ELISA for LOX-PP with purified antibody showing the affinity of the raised antibody with purified LOX-PP protein. (**f**) SDS-PAGE of purified LOX-PP antibody using two different volumes (Lane-1: 2.5 μl, Lane-2: 5.0 μl) and stained with coomassie stain to show heavy chain (HC) at 55 kDa and light chain (LC) at 25 kDa.

The pQE 30Xa + LOX-PP expressed in M15 (pREP4) *E*.*coli* cells was purified using Ni-NTA agarose columns (Fig. 1a). The purified protein was confirmed by western blot analysis before and after his tag cleavage with an anti-LOX-PP and anti-His tag antibody (Fig. 1b,c). The His - tag cleaved LOX-PP was also confirmed by mass spectrometry (Fig. 1d) and the purified protein was used for antibody production. Direct ELISA for LOX-PP using the in house purified antibody showed the specificity for LOX-PP protein as measured by antibody titration (Fig. 1e,f).

### Overexpression of LOX-PP in HUVECs

**Figure 2:**
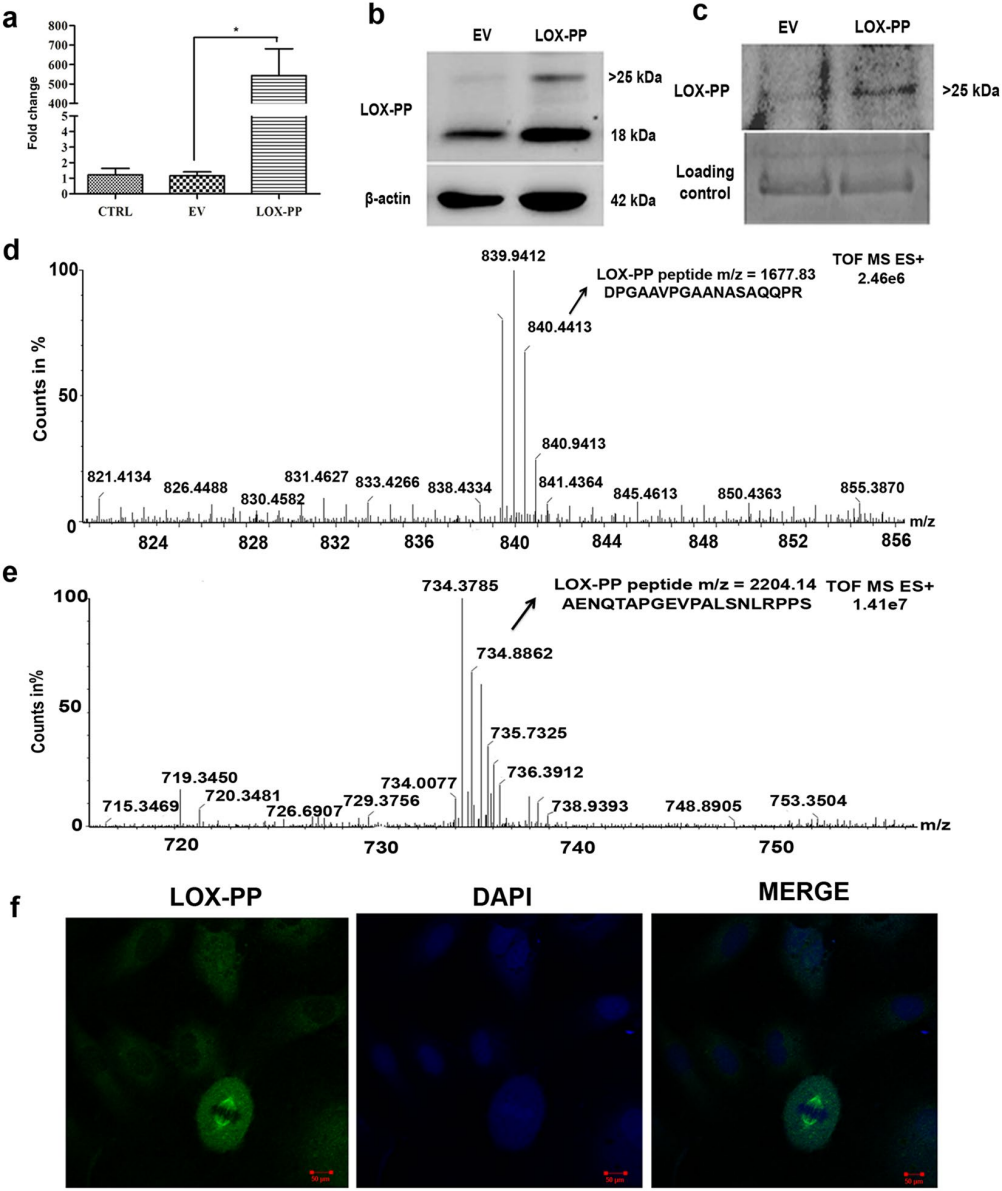
Transient transfection of LOX-PP, confirmation by western blot and mass spectrometry: (**a**) mRNA expression of LOX-PP overexpressed in HUVECs after 48 h (**b**) After 48 h of transfection, cell extracts were collected and probed using LOX-PP antibody by western blot and was normalized with β- actin (EV- empty vector, LOX-PP-LOX-PP transfected cells). The corresponding full-length blots are represented in Supplementary Figs 3 and 4. (**c**) Conditioned medium was collected and it was immunoprecipitated using LOX-PP antibody, immunoprecipitated samples were subjected to western blot using antibody against LOX-PP and coomassie staining was considered as loading control, the corresponding full-length blots are represented in Supplementary Figs 5 and 6. (**d**) Mass spectrum of LOX-PP in cell lysate. (**e**) Mass spectrum of LOX-PP in conditioned medium. (**f**) Immunofluorescence for LOX-PP in HUVECs after overexpression (Magnification 63x).Cells were stained with Alexa 488 and counterstained with DAPI.

Overexpression of LOX-PP with pcDNA 3.1/His A + LOX-PP construct in HUVECs was confirmed at RNA level (Fig. 2a). No cell toxicity was observed by MTT (Fig. S2a) with a maximum expression seen at 48 h post-transfection (Fig. S2b) and this time point was used for subsequent experiments. LOX-PP overexpression was confirmed at protein level by Western blot in HUVECs extracts (Fig. 2b). Two bands, one at 18 kDa which corresponds to the non-glycosylated form and another (>25 kDa), the N- glycosylated form of LOX-PP were observed in the cell extracts^22^. Immunoprecipitation of the conditioned medium showed (Fig. 2c) a band of >25 kDa which corresponds to the N-glycosylated form^23^. LOX-PP overexpression was further confirmed by mass spectrometry in cell lysate and conditioned medium (Fig. 2d,e) which showed high intensity peptides with a mass of 1677.83 (DPGAAVPGAANASAQQPR) and 2204.14 (AENQTAPGEVPALSNLRPPS) which was identified as LOX-PP protein coverage by Protein Link Global Server (PLGS). Peptides relevant to LOX-PP were not identified in empty vector (EV) transfected and control (CTRL) HUVECs. In HUVECs, LOX-PP overexpression was found to be localized to the nucleus (spindle fibers) and cytoplasm by immunofluorescence (Fig. 2f).

### LOX-PP down regulated angiogenesis signaling pathways in HUVECs

**Figure 3:**
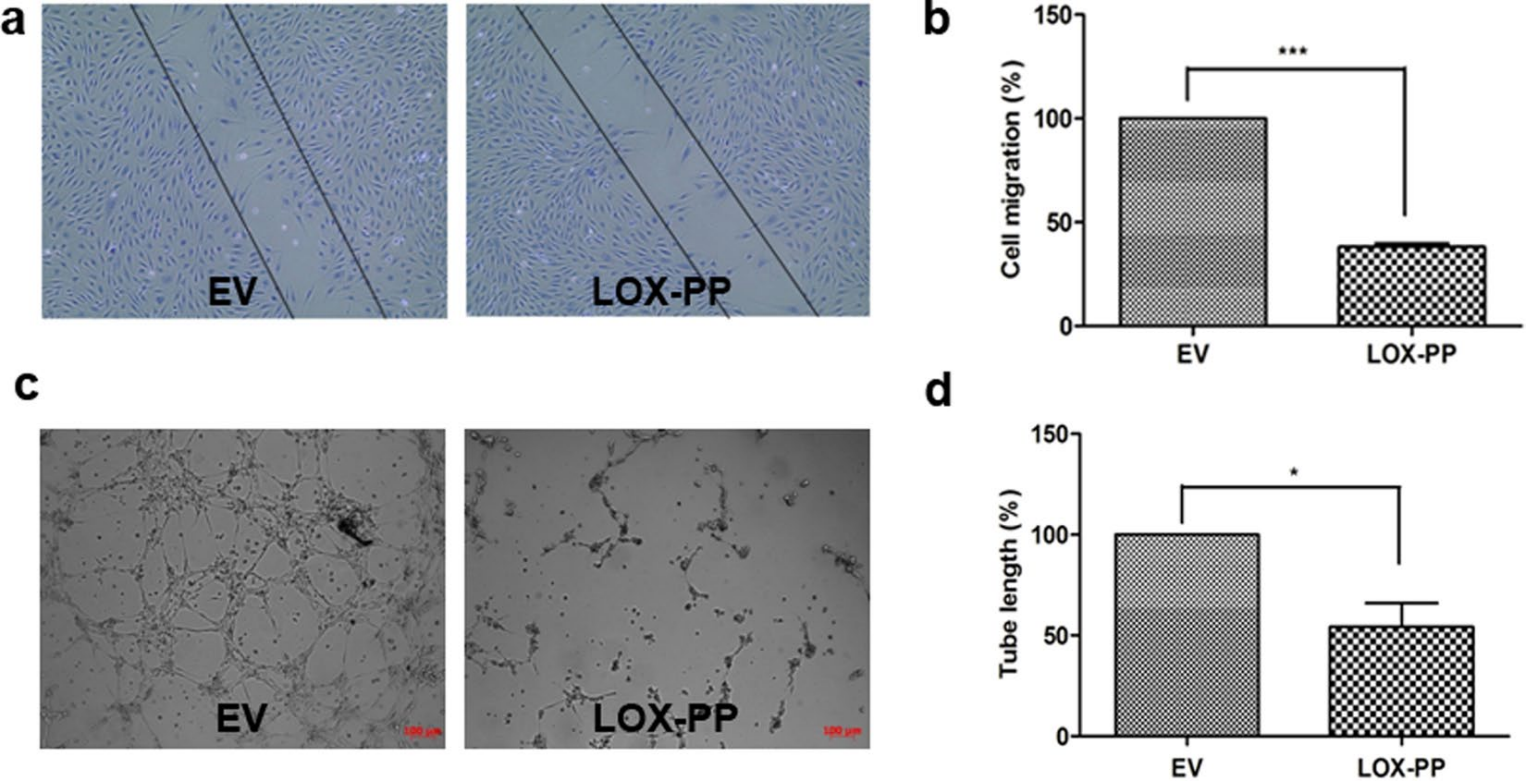
Effect of LOX-PP overexpression on HUVEC’s migration and tube formation: Overexpression of LOX-PP inhibits (**a**) cell migration and (**b**) Bar graph represents the quantification of scratch assay (**c**) tubule formation (**d**) Bar graph represents the quantification of tube formation assay. Values were expressed as mean ± SD, n = 3. ***p < *0*.*001*, *p < *0*.*05* versus EV. (EV – Empty vector).

**Figure 4:**
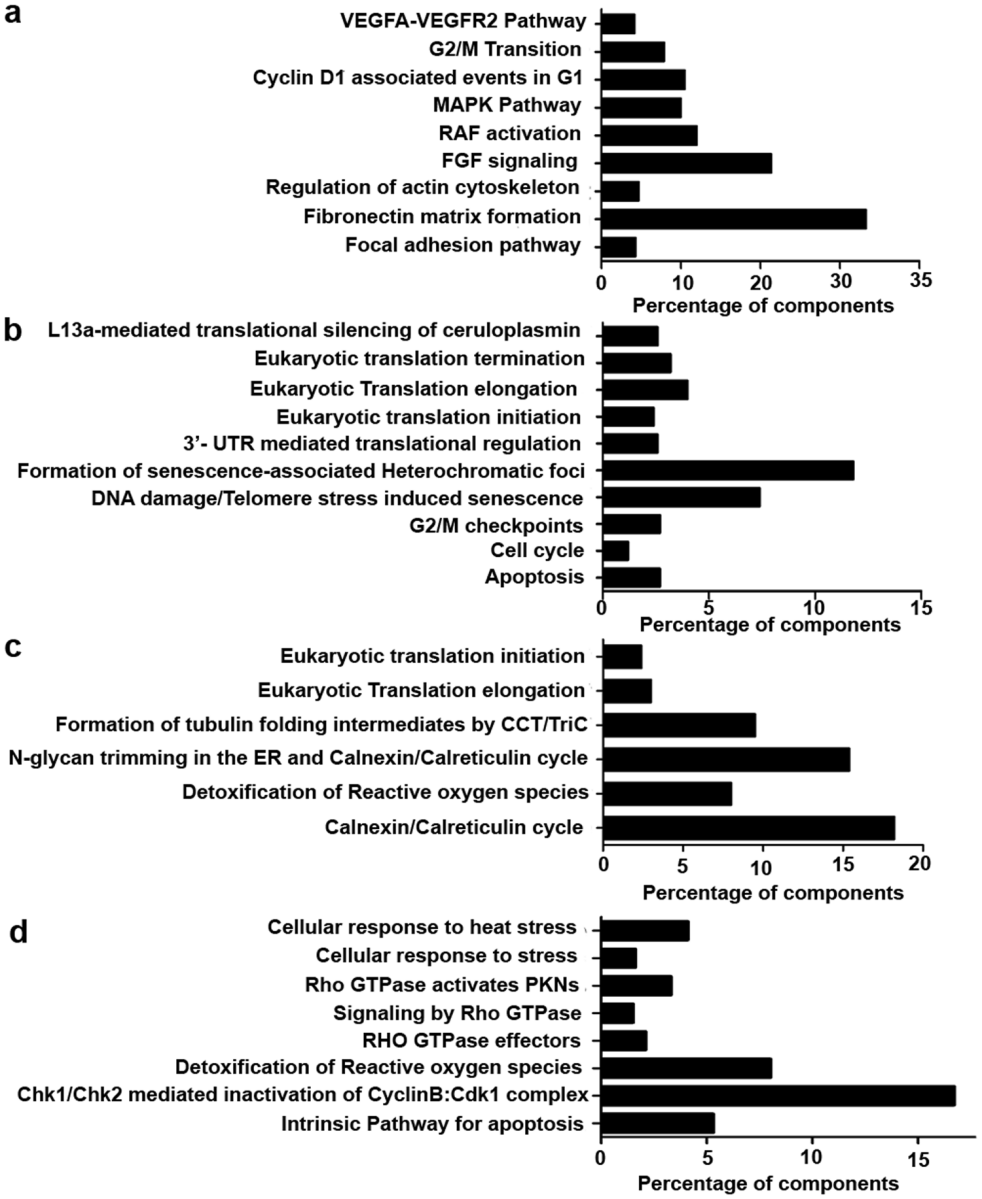
Proteomic analysis of LOX-PP overexpression in HUVECs: (**a**) Reactome and KEGG pathway analysis of the 372 uniquely expressed proteins in empty vector by ConsensusPathDB online tool (http://cpdb.molgen.mpg.de/) with *p* < 0.01, which showed components of VEGF signaling, MAPK signaling, RAF signaling and FGF signaling to be significantly increased. (**b**) Reactome and KEGG pathway analysis of the 75 uniquely expressed proteins in LOX-PP overexpressed cells by ConsensusPathDB online tool (http://cpdb.molgen.mpg.de/) with *p* < 0.01, which showed components of apoptosis, cell cycle and G2M check points to be increased. (**c**) Reactome and KEGG pathway analysis of proteins that are downregulated by LOX-PP overexpression. (**d**) Reactome and KEGG pathway analysis of proteins that are upregulated by LOX-PP overexpression.

Initially, HUVECs overexpressed with LOX-PP showed significant reduction in cell migration (Fig. 3a,b) and tube formation (Fig. 3c,d). To further understand this function, LOX-PP overexpressed HUVEC lysates were subjected to mass spectrometry. The LC-MS^e^ data processed by PLGS identified a total of 815 proteins in EV and LOX-PP overexpressed HUVECs. About 372 and 75 proteins were identified exclusively in EV and LOX-PP overexpressed HUVEC cells respectively. Whereas 184 proteins were found to be common between them. These proteins were annotated using the ConsensusPathDB online tool (http://cpdb.molgen.mpg.de/). The 372 proteins which were detected in EV expressed HUVECs were found to be involved in VEGF signaling, FGF signaling, MAPK pathway, RAF signaling, actin cytoskeleton pathway and fibronectin mediated pathways (Fig. 4a) which were notably absent in LOX-PP overexpressed cells. The 75 proteins which were detected in LOX-PP overexpressed cells were found to be involved in apoptosis, senescence and cell cycle checkpoints (Fig. 4b). The 184 proteins which were commonly present in EV and LOX-PP with a relative quantification value of >2 fold and <2 fold were annotated (Fig. 4c,d) as upregulated and downregulated proteins using the online tool ConsensusPathDB (http://cpdb.molgen.mpg.de/). This showed that the intrinsic pathways for apoptosis, stress regulators and G2M DNA check point damage proteins were upregulated and actin, tubulin folding, calnexin- clareticulin signaling and ephrin signaling pathways were downregulated in LOX-PP expressed cells. Since LOX-PP overexpressed HUVECs showed down regulation of numerous angiogenesis related pathways, we further performed *in vitro* functional assays to confirm this observation by stimulating the HUVECs with VEGF as pro-angiogenic factor.

### LOX-PP inhibits cell proliferation

**Figure 5:**
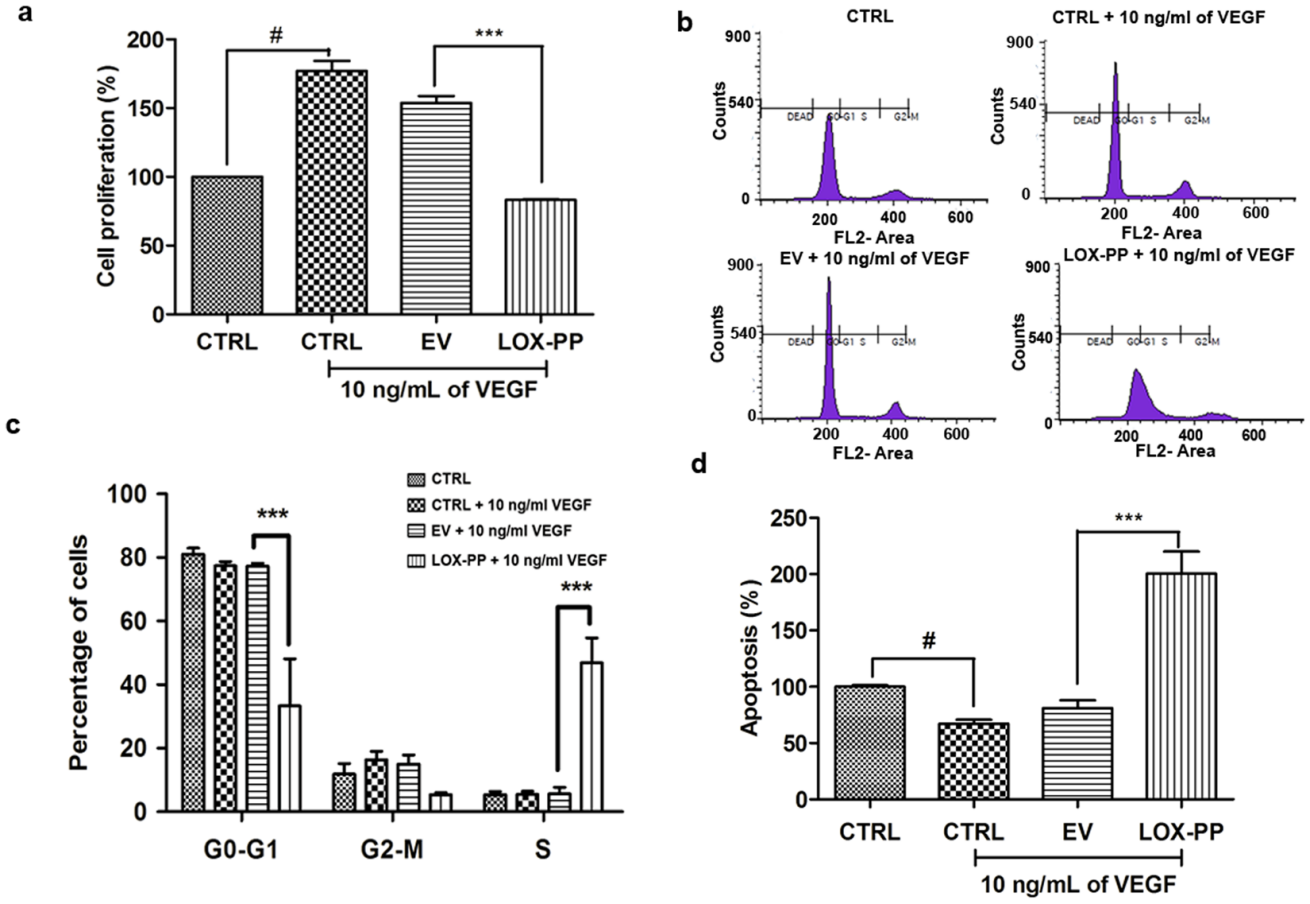
Effect of LOX-PP on VEGF induced proliferation and cell cycle in HUVECs: (**a**) Bar graph represents the quantification of proliferation assay using BrdU ELISA kit. (**b**) Propidium iodide staining and flow cytometry analysis was performed to determine the percentage of cells in G1, S and G2 phase of cell cycle. (**c**) Bar graph represents the quantification of cell cycle analysis. (**d**) Bar graph represents the quantification of apoptosis assay using Cell death ELISA kit. Values were expressed as mean ± SD, n = 3. ***p < *0*.*001* versus VEGF + EV. ^#^*p* < *0*.*05* versus CTRL. (CTRL – Control; EV – Empty vector).

As shown in Fig. 5a, HUVECs which were treated with VEGF showed increased cell proliferation which was significantly (*p* = *0*.*001*) inhibited by LOX-PP overexpression. Cell cycle analysis indicated that the cells were arrested in S phase leading to decreased cell entry into G2-M phase (Fig. 5b,c) thereby inhibiting cell proliferation. These results substantiated the inference of our proteomic analysis which showed upregulation of G2-M checkpoint proteins in LOX-PP overexpressed HUVECs (Fig. 4b). The EV transfected cells did not have any effect and was similar to that of control cells.

### LOX-PP induces apoptosis

As shown in Fig. 5d, LOX-PP significantly increased apoptosis by 50% more than the EV transfected cells in the presence of 10 ng/mL of VEGF. HUVECs which were treated with VEGF showed reduced apoptosis which was significantly (*p* = *0*.*001*) increased by LOX-PP overexpression as measured by the apoptosis assay.

### LOX-PP inhibits cell migration

**Figure 6:**
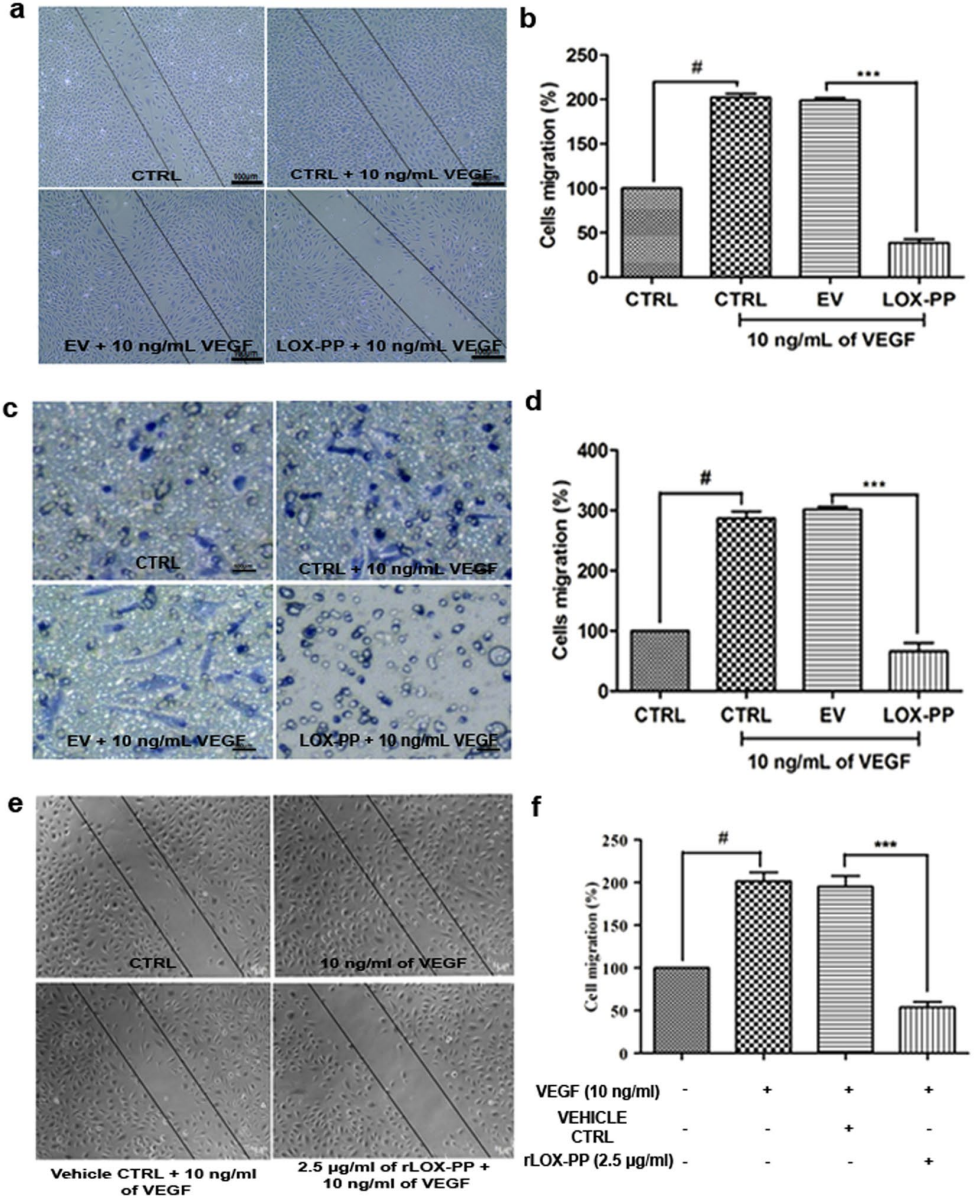
Effect of LOX-PP on VEGF induced cell migration in HUVECs: (**a**) For scratch assay HUVEC cells were induced with 10 ng/mL of VEGF, 10 ng/mL of VEGF + EV and 10 ng/mL of VEGF + LOX-PP for 18 h and were imaged using Axio observer microscope at 5x magnification. (**b**) Bar graph represents the quantification of scratch assay. (**c**) For transwell migration assay HUVEC cells were induced with 10 ng/mL of VEGF, 10 ng/mL of VEGF + EV and 10 ng/mL of VEGF + LOX-PP for 8 h and were imaged using Axio observer microscope at 5x magnification. (**d**) Bar graph represents the quantification of transwell migration assay. Cells were counted using ImageJ software (expressed as % of controls). (**e**) HUVEC cells were induced with 10 ng/mL VEGF, 10 ng/mL VEGF + Vehicle ctrl and 10 ng/ml of VEGF + 2.5 μg/ml of rLOX-PP for 18 h and were imaged using Axio observer microscope at 5 x magnification. (**f**) Bar graph represents the quantification of scratch assay. Values were expressed as mean ± SD, n = 3. ***p < *0*.*001* versus VEGF + EV. ***p < *0*.*001* versus VEGF + Vehicle ctrl. #*p* < *0*.*05* versus CTRL. (CTRL – Control; EV – Empty vector; Vehicle ctrl – Vehicle control).

The crucial event of angiogenesis is the directional migration of endothelial cells. HUVECs treated with VEGF displayed increased cell migration when compared to CTRL cells. The scratch assay (Fig. 6a) and transwell migration assays (Fig. 6c) showed that LOX-PP overexpressed HUVECs significantly inhibited cell migration by 81.18% (*p* = 0.001; Figs. 6b) and 77.28% (*p* = 0.0002; Fig. 6d) respectively. This validated our finding in proteomic analyses, where LOX-PP overexpression down regulated proteins involved in actin tubulin folding and cytoskeletal regulated pathways (Fig. 4c). The EV transfected cells did not have any effect and was similar to that of control cells. Furthermore, HUVECs treated with 2.5 μg/mL of rLOX-PP also significantly decreased the cell migration (Fig. 6e,f; *p* = *0*.*001)*.

### LOX-PP inhibits cell attachment

**Figure 7:**
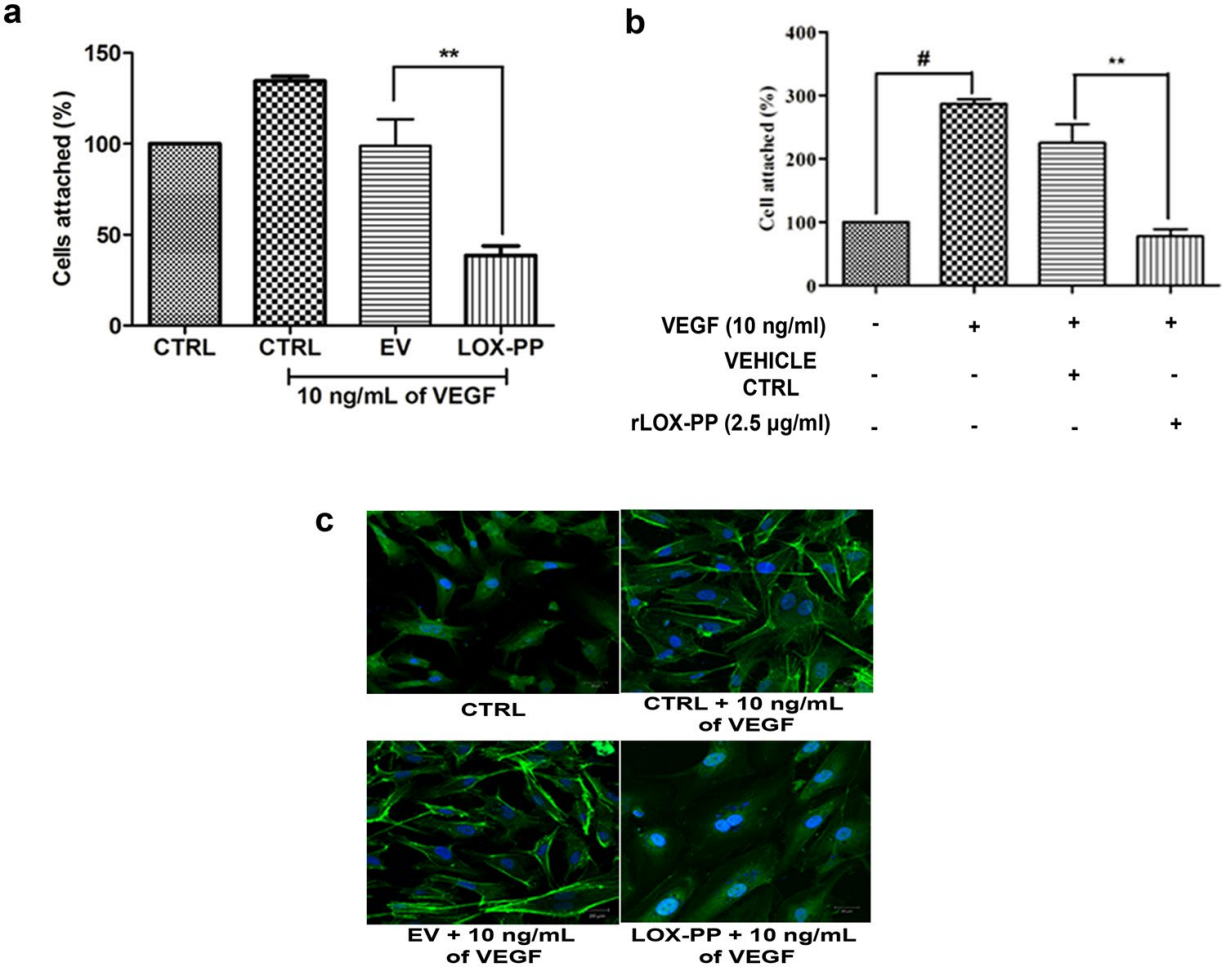
Effect of LOX-PP on VEGF induced cell attachment and reorganization of F-actin: (**a**) Bar graph represents the quantification of cell attachment after overexpression of LOX-PP, attached cells were counted using ImageJ software (expressed as % of controls). (**b**) Bar graph represents the quantification of cell attachment after addition of rLOX-PP, attached cells were counted using ImageJ software (expressed as % of controls). (**c**) Inhibitory effect of overexpressed LOX-PP on the reorganization of actin cytoskeleton induced by VEGF (10 ng/mL) under LSM 880 confocal laser scanning microscope. HUVECs were fixed and stained for filamentous actin with phalloidin FITC conjugate. Cells were also transfected with EV. Nucleus is counter-stained with DAPI. Values were expressed as mean ± SD, n = 3. **p < *0*.*01* versus VEGF + EV. **p < *0*.*01* versus VEGF + Vehicle ctrl. ^#^p < *0*.*05* versus CTRL. (CTRL – Control; EV – Empty vector; Vehicle ctrl – Vehicle control).

Endothelial cell attachment and adhesion to extracellular matrix are major processes for the progression of angiogenesis. Figure 7a shows that VEGF treated HUVECs showed increased cell attachment to the extra cellular matrix. In contrast to this LOX-PP transfected HUVECs significantly (*p* = *0*.*03*) inhibited (68%) cell attachment compared to EV transfected cells. Interestingly addition of 2.5 μg/ml of rLOX-PP also significantly decreased the cell attachment (Fig. 7b; *p* = *0*.*01*).

### LOX-PP decreased F-actin organization

Treatment with VEGF resulted in a substantial increase in the number of stress fibers and rearrangement of actin organization aiding in directional migration. Whereas LOX-PP overexpressed cells attenuated actin organization and stress fiber formation compared to VEGF stimulated EV transfected cells as seen in Fig. 7c. The EV transfected cells did not have any effect and was similar to that of control cells.

### LOX-PP inhibits tube formation

**Figure 8:**
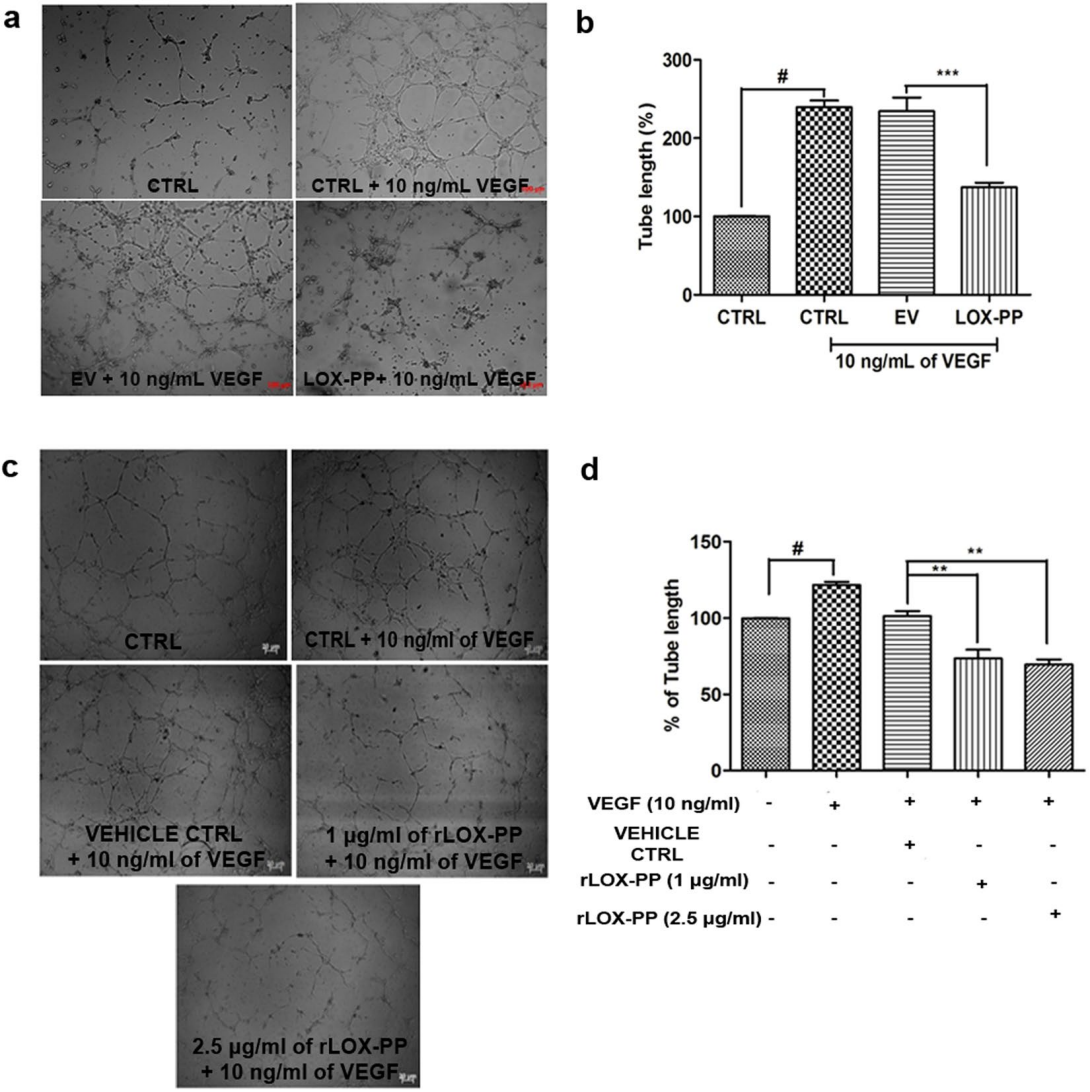
Effect of LOX-PP on VEGF induced tube formation in HUVECs: (**a**) HUVEC cells were induced with 10 ng/mL VEGF, 10 ng/mL of VEGF + EV and 10 ng/mL of VEGF + LOX-PP for 4 h and were imaged using Axio observer microscope at 5x magnification. (**b**) Bar graph represents the quantification of tube length measured using ImageJ software (expressed as % of controls). (**c**) HUVEC cells were induced with 10 ng/mL VEGF, 10 ng/mL VEGF + Vehicle ctrl, 10 ng/mL of VEGF + 1.0 μg/ml of rLOX-PP and 10 ng/ml of VEGF + 2.5 μg/ml of rLOX-PP for 4 h and were imaged using Axio observer microscope at 5x magnification. (**d**) Bar graph represents the quantification of tube length measured using ImageJ software (expressed as % of controls). Values were expressed as mean ± SD, n = 3. ***p < *0*.*001* versus VEGF + EV. **p < 0.01 versus VEGF + Vehicle ctrl. ^#^*p* < 0.05 versus CTRL. (CTRL – Control; EV – Empty vector; Vehicle ctrl – Vehicle control).

The endothelial cells form capillary loop like structure in response to angiogenic signals. HUVECs treated with VEGF showed significant increase in tube length, when compared to CTRL. However, this was significantly inhibited in LOX-PP (42.45%; *p* = *0*.*0005*) overexpressed cells (Fig. 8a,b). This correlated with our proteomic analysis where the fibronectin matrix formation pathway seen in EV was not found in LOX-PP overexpressed cells (Fig. 4a,b). No significant reduction in tube length was observed in VEGF stimulated EV transfected HUVECs. This observation was further confirmed by treating HUVECs with rLOX-PP which significantly diminished the tube length *(p* = *0*.*01)*. (Fig. 8c,d). These results indicate that inhibition of angiogenesis events is not only achieved by overexpressing LOX-PP but also by addition of rLOX-PP.

### LOX-PP inhibits FAK and ERK phosphorylation

**Figure 9:**
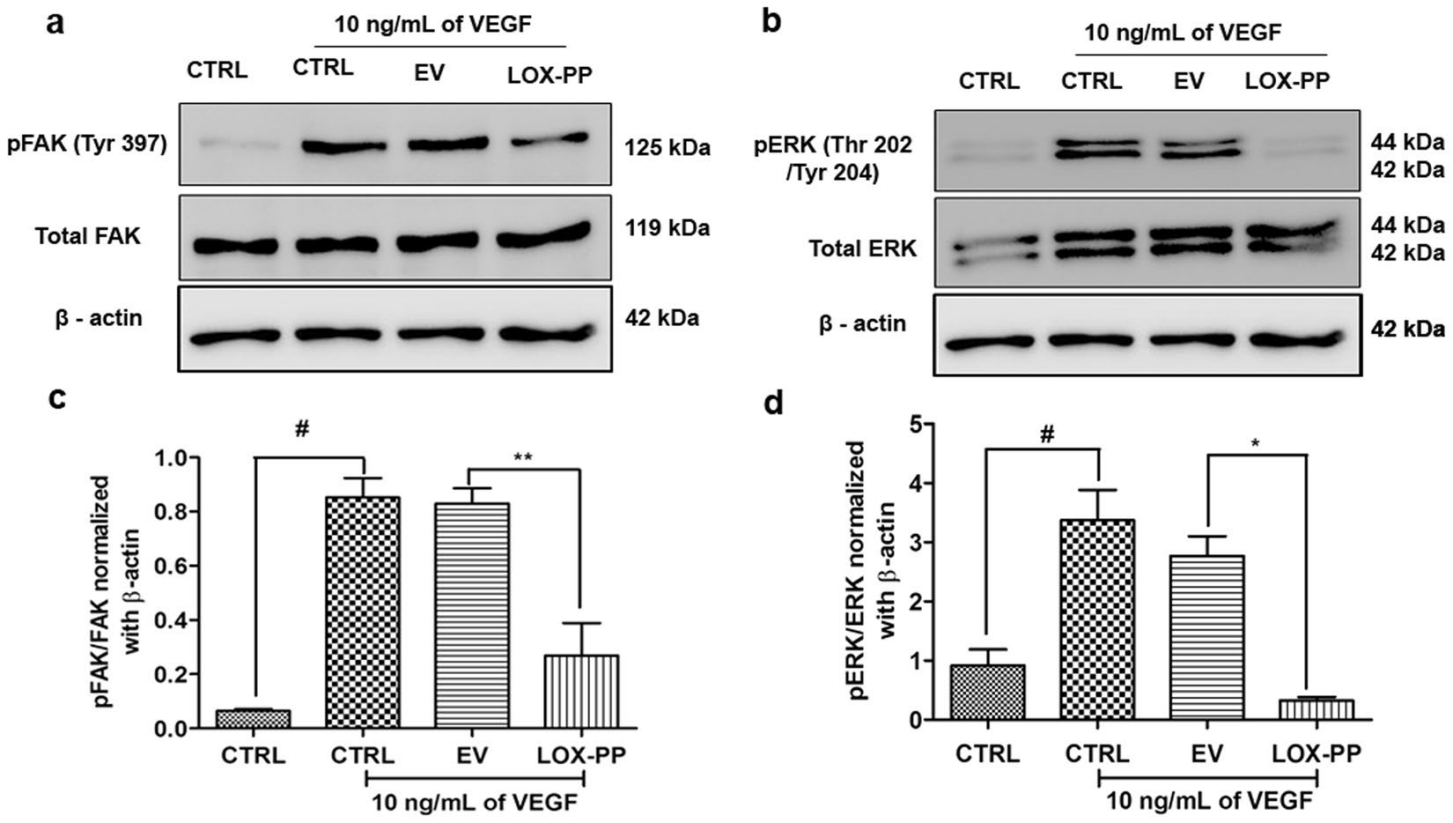
Inhibitory effect of LOX-PP on FAK (Tyr397) and ERK (Thr202/Tyr204) phosphorylation induced by VEGF in HUVECs: (**a**) HUVECs were transfected with LOX-PP and EV. After transfection, cells were treated with 10 ng/mL of VEGF for 15 min. Whole cell extract were prepared and the samples were subjected to western blotting using antibodies against pFAK (Tyr397), total FAK and β–actin was used as loading control. The full-length blots are represented in Supplementary Figs 7, 8 and 11. (**b**) HUVECs were transfected with LOX-PP and EV. After transfection, cells were treated with 10 ng/mL of VEGF for 15 min. Whole cell extract were prepared and the samples were subjected to western blotting using antibodies against pERK (Thr202/Tyr204), total ERK and β–actin was used as loading control. The full-length blots are represented in Supplementary Figs 9, 10 and 11. (**c**) Bar graph represents quantification of the western blot using ImageJ software for FAK (Tyr397). (**d**) Bar graph represents quantification of the western blot using ImageJ software for ERK (Thr202/Tyr204). Values were expressed as mean ± SD, n = 3. **p < *0*.*01*, *p < *0*.*05* versus VEGF + EV. ^#^*p* < *0*.*05* versus CTRL. (CTRL – Control; EV – Empty vector).

**Figure 10:**
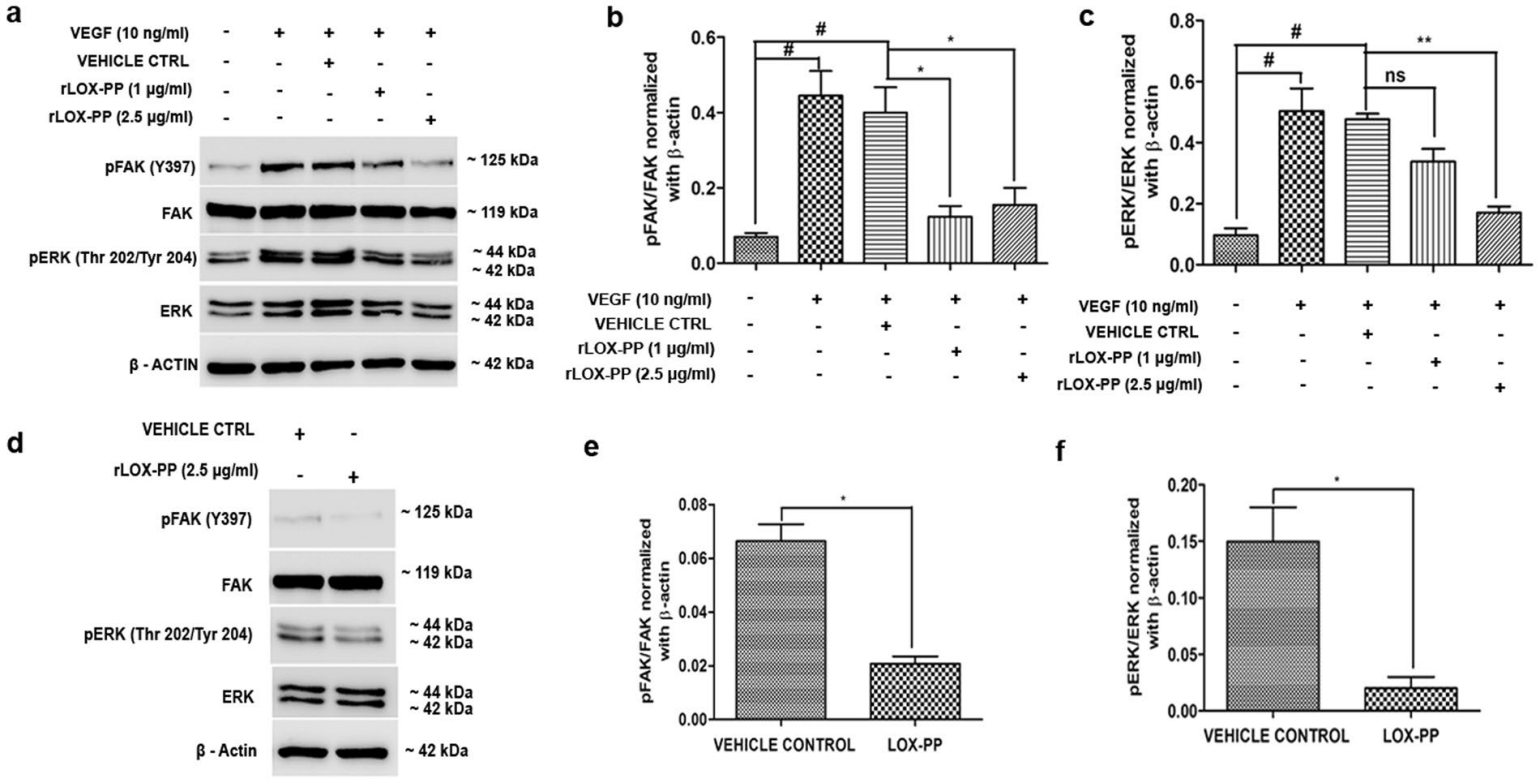
Effect of rLOX-PP on FAK (Tyr397) and ERK (Thr202/Tyr204) phosphorylation induced by VEGF in HUVECs: (**a**) HUVECs were treated with rLOX-PP and vehicle ctrl. After treatment, cells were treated with 10 ng/mL of VEGF for 15 min. Whole cell extract were prepared and the samples were subjected to western blotting using antibodies against pFAK (Tyr397), total FAK, pERK (Thr202/Tyr204), total ERK and β–actin was used as loading control. The full-length blots are represented in Supplementary Figs 12, 13, 14, 15 and 16. (**b**) Bar graph represents the quantification of western blot using ImageJ software for pFAK (Tyr397) and (**c**) pERK (Thr202/Tyr204). (**d**) HUVECs were treated with rLOX-PP without stimulation of VEGF. Cell lysate were subjected to western blotting using pFAK (Tyr397), total FAK, pERK (Thr202/Tyr204), total ERK and β–actin. The full-length blots are represented in Supplementary Figs 12, 13, 14, 15 and 16. (**e**) Densitogram of the western blot shows the ratio of pFAK to total FAK, normalized with β-actin. (**f**) Densitogram of the western blot shows the ratio of pERK to total ERK, normalized with β-actin. Values were expressed as mean ± SD, n = 3. **p < 0.01, *p < *0*.*05* versus VEGF + Vehicle ctrl. ^#^*p* < *0*.*05* versus CTRL. (CTRL – Control; Vehicle ctrl – Vehicle control).

Focal adhesion kinase is a major component of the focal adhesion complex that aids in attachment of cells to the extracellular matrix and plays an essential role in cell adhesion, survival, migration and cell cycle control. We examined the phosphorylation of FAK protein at Tyrosine 397 which is essential for its activation. As shown in Fig. 9a, LOX-PP overexpression significantly reduced (*p* = *0*.*009;* Fig. 9c) VEGF stimulated FAK phosphorylation at Tyr397, compared to the EV. Furthermore, VEGF stimulated ERK phosphorylation (Fig. 9b) at Thr202/Tyr204 was also significantly reduced (*p* = *0*.*02*; Fig. 9d). No significant change was observed in EV transfected cells. This was further confirmed by adding rLOX-PP to HUVECs (Fig. 10a) which significantly decreased pFAK (*p* = *0*.*05*; Fig. 10b) and pERK (*p* = *0*.*01;* Fig. 10c). Cells treated with rLOX-PP (Fig. 10d) significantly decreased pFAK (*p* = *0*.*05*; Fig. 10e) and pERK (*p* = *0*.*01*; Fig. 10f) levels even in the absence of VEGF stimulation suggesting its direct role in downregulating angiogenesis signaling.

### rLOX-PP reduced vascular network in chicken chorioallantoic membrane

**Figure 11:**
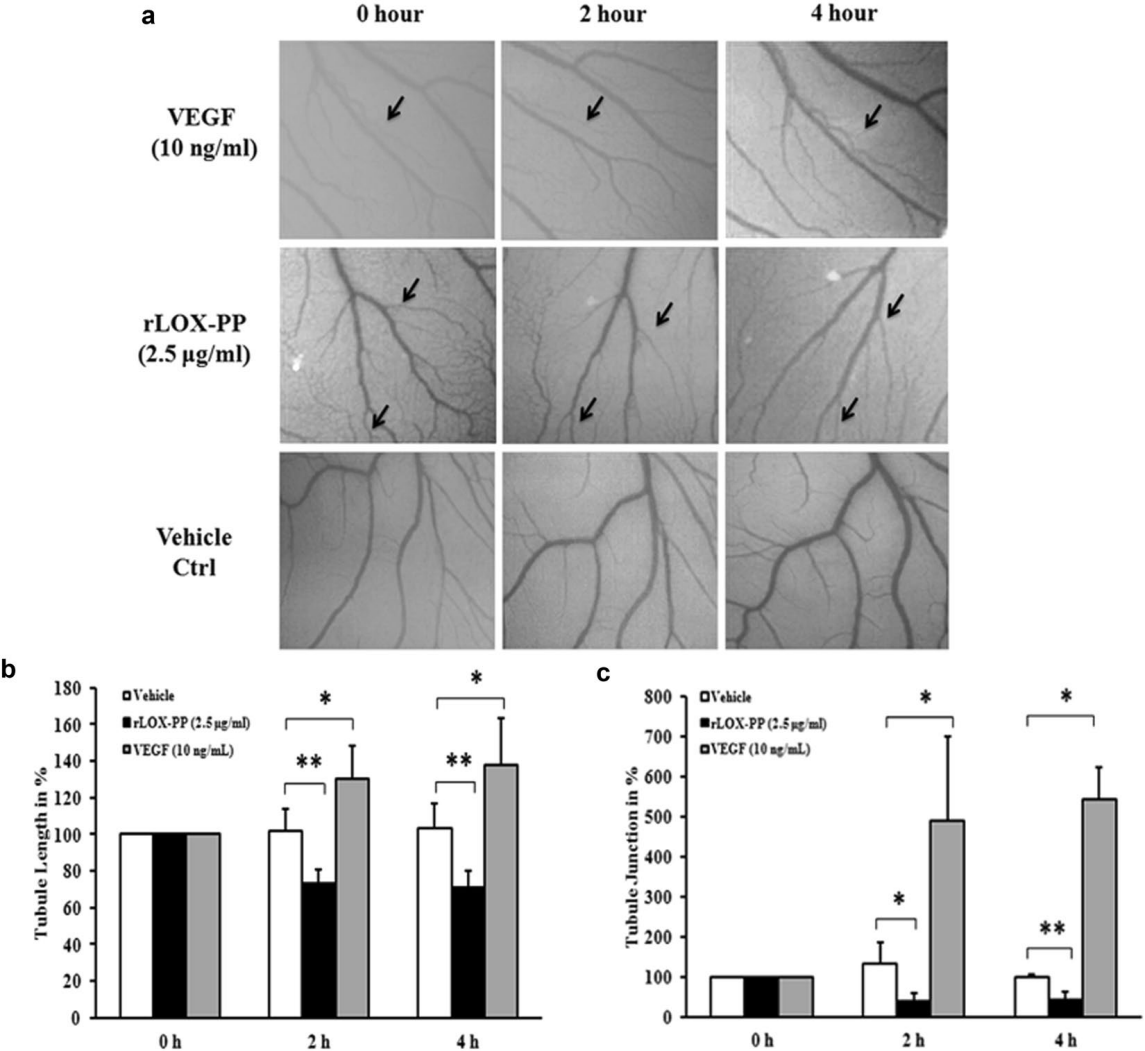
Effect of rLOX-PP in *in vivo* angiogenesis CAM assay: (**a**) Representative photographs showing the rLOX-PP (2.5 μg/ mL) treated CAM, vehicle ctrl treated CAM and 10 ng/ml of VEGF treated CAM (positive control) at 0 h, 2 h and 4 h. (**b**) Quantification of the blood vessels in terms of measuring tubule length and (**c**) tubule junction. Vessel length and junction were noted (indicated in arrow) in each CAM and changes are shown in percentage in relation to the vehicle ctrl. Values are expressed as mean ± SD, n = 3. **p < *0*.*01* and *p < *0*.*05* versus Vehicle ctrl. *p < *0*.*05* versus 10 ng/mL of VEGF. (Vehicle ctrl – Vehicle control).

To confirm the above observations we examined the anti-angiogenic effect of rLOX-PP by performing the chicken chorioallantoic membrane (CAM) assay. Topical administration of VEGF increased tubule length and tubule junction (Fig. 11a) whereas addition of rLOX-PP (2.5 μg/ml) induced changes in the structure of the vascular network in terms of reduced tubule length (Fig. 11b; *p* = *0*.*01*) and tubule junction (Fig. 11c; *p* = *0*.*01*). The regular structure of the vascular network was disrupted and the length of blood vessels was decreased by rLOX-PP compared to vehicle control which showed regular network of blood vessels that consisted of continuously perfused arteries and veins with adjacent capillary bed.

### Uptake of rLOX-PP in HUVECs showing intracellular localization

**Figure 12:**
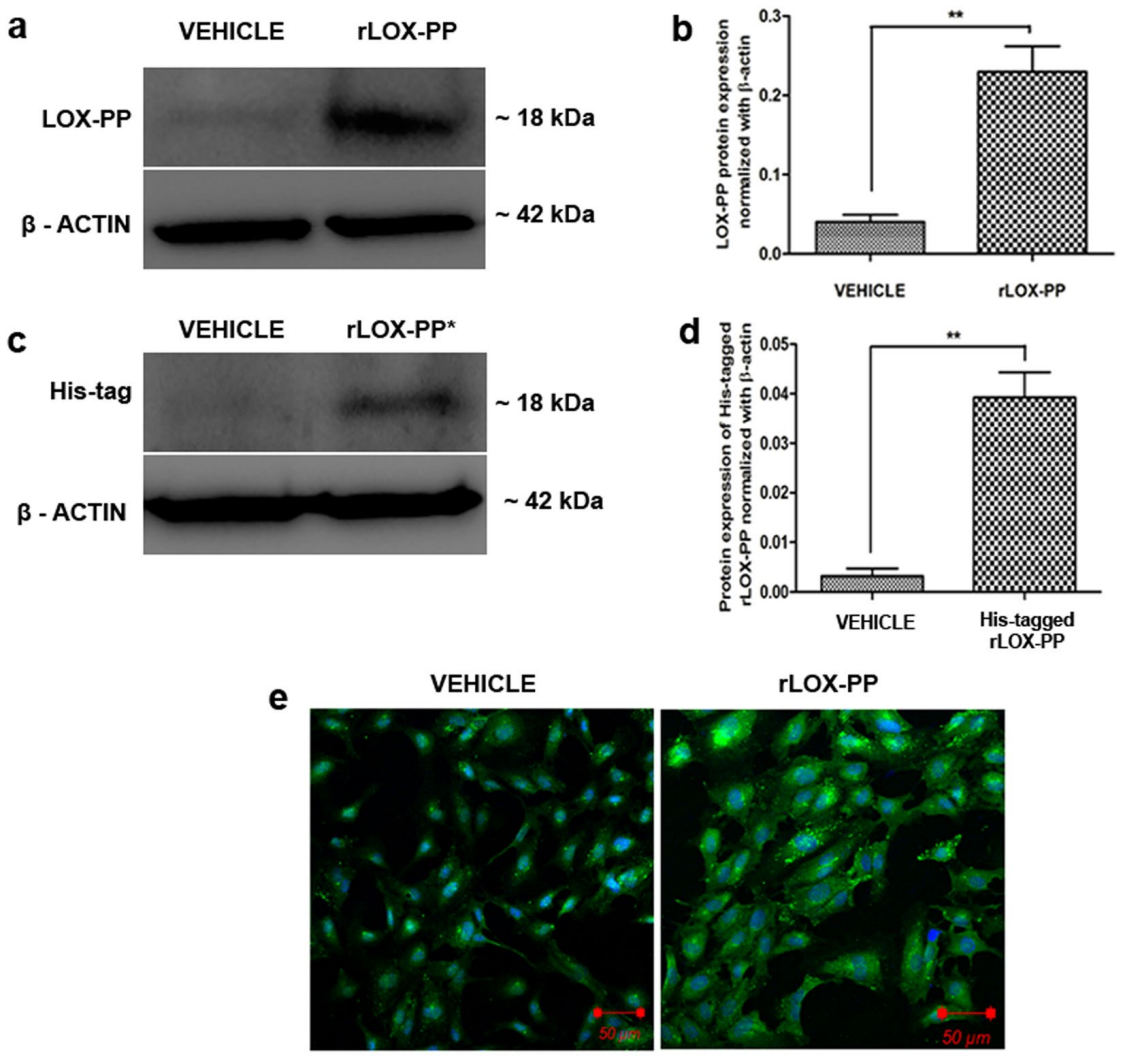
Uptake and localization of rLOX-PP in HUVECs. (**a**) LOX-PP protein expression was increased upon addition of rLOX-PP in HUVECs when compared with vehicle ctrl. The full-length blots are represented in Supplementary Figs 17 and 18. (**b**) Densitogram of the western blot shows LOX-PP protein normalized with β-actin. (**c**) Western blot for His-tag indicates rLOX-PP with His-tag which was added exogenously to HUVECs was found in the cell lysate indicating its uptake. The full-length blots are represented in Supplementary Figs 19 and 20. (**d**) Densitogram of the western blot shows His-tagged LOX-PP protein expression normalized with β-actin. Values were expressed as mean ± SD, n = 3. **p < *0*.*01* versus vehicle ctrl. (Vehicle ctrl – Vehicle control). (**e**) Increased LOX-PP expression is observed upon addition of rLOX-PP to HUVECs under LSM 880 confocal laser scanning microscope. Cells were stained with Alexa 488 and counterstained with DAPI.

In order to address if rLOX-PP is taken up by the HUVECs to exert this function, we examined its uptake by incubating the cells with 2.5 µg/ml of rLOX-PP (with and without His tag) for 48 h after which the cells were lysed and subjected to western blot. The blots indicated elevated expression of LOX-PP (Fig. 12a,b) and His tag in the rLOX-PP (with His tag) treated cells (Fig. 12c,d). In addition, immunofluorescence displayed the localization of rLOX-PP with higher intensity in the cytoplasm compared to vehicle control (Fig. 12e). We ensured that the added rLOX-PP was washed well before proceeding for localization and lysate preparation for western blot. This data demonstrated the uptake of rLOX-PP by HUVECs which was localized in the cytoplasm.

## Discussion

LOX has been reported to have a paradoxical role in tumor biology^24^. Interestingly, the tumor suppressor activity of LOX was mapped to the propeptide region of the protein and thus LOX-PP was considered as an attractive anti-angiogenesis candidate molecule^14^. In the present study we used HUVECs, as a model system for angiogenesis and by several experimental approaches we have assessed the functional role of LOX-PP. The results indicate that LOX-PP overexpression down regulates major angiogenesis signaling pathways and attenuated cell proliferation, migration, adhesion, thereby demonstrating its anti-angiogenic potential. Previous reports indicate that recombinant LOX-PP inhibits RAS signaling in pancreatic, breast and prostate cancers in nude mice^15,19,25^. Interestingly, we found that addition of rLOX-PP to endothelial cells inhibits functional events of angiogenesis in HUVEC. Zhao *et al*. reported that LOX-PP attenuates fibronectin mediated Her-2/neu-driven breast cancer cell migration^26^. Apart from its role in transformed cells, LOX-PP was also found to be accumulated in the endothelium of injured arteries acting as a feedback inhibitor of TNF-alpha mediated vascular smooth muscle proliferation and MMP-9 synthesis by down regulating ERK-1/2 MAP kinase pathway^22^.

Our proteomic analysis reveals that LOX-PP likely functions by down regulating VEGF, MAPK, FGF, focal adhesion, fibronectin mediated matrix formation and cytoskeleton regulation pathway which are the key mediators of angiogenesis. Bais *et al*. have reported that rLOX-PP can reduce the expression of proliferation markers and induce apoptosis in xenograft models^27^. Similarly overexpression of LOX-PP was also found to inhibit cell proliferation, arrest cells at S phase in the cell cycle and increased apoptosis in HUVECs. LOX-PP also inhibited the migration of endothelial cells, cell adhesion and tube length significantly in both overexpressed/rLOX-PP treated HUVECs thereby preventing the angiogenesis process.

VEGF stimulated endothelial cell migration is executed by phosphorylating FAK^28,29^. Autophosphorylation at Tyr-397 is essential for FAK function, which is apparent from its role in integrin engagement, G-protein coupled receptor activation, growth factor receptor stimulation and accumulation of actin stress fibers. We observed that the crucial FAK phosphorylation was downregulated in rLOX-PP treated and LOX-PP overexpressed HUVECs with a concomitant decrease in F-actin content. Autophosphorylation of FAK increases its affinity to bind with various proteins possessing SH2 domains, which thereby leads to phosphorylation of other tyrosine residues like ERK which are involved in regulation of cell differentiation, proliferation and apoptosis^30,31^. LOX-PP is known to interact with HSP70 and c-Raf, thereby reducing ERK activation leading to reduced downstream activation of NF-kB, migration and anchorage-independent growth^32^. This study signified that both LOX-PP overexpression/rLOX-PP addition reduced phosphorylated ERK1/2 and thereby arresting cells in S phase impeding HUVECs angiogenesis process.

Morgan *et al*. has shown localization of overexpressed LOX-PP in cytoplasm, trans-golgi network and as well as in the nucleus of lung cancer cells^33^. Guo *et al*. localized rLOX-PP in the cytoplasm of differentiating osteoblast where it associates with the microtubule network^23^. With LOX-PP being reported at multiple cellular locations we speculated that it can execute its function in cytoplasm and as well as in the nucleus. Interestingly we found that overexpression of LOX-PP was found to be localized to the nucleus (spindle fibers) and cytoplasm. (Fig. 2f). The mode of cell uptake of LOX-PP is still elusive with Ozdner *et al*. reporting a PI3K‐dependent macropinocytosis in endosomes which facilitate its uptake into the cytoplasm. We have demonstrated that rLOX-PP is taken up by the endothelial cells and is localized in cytoplasm (Fig. 12e). Does LOX-PP enter the cell through a PI3K-dependent macropinocytosis^34^ and down regulates the downstream VEGF signaling events or does it exert its function extracellularly by binding to VEGF receptor or does it act at the level of DNA^35^ is currently not addressed and needs to be further delved. We have demonstrated the anti-angiogenic effect of LOX-PP in VEGF mediated angiogenesis, however further studies are warranted to know its function with other angiogenic stimulants.

This study provides evidence that functionally LOX-PP can mitigate each step in the dynamic process of angiogenesis when stimulated with VEGF and as well as in unstimulated HUVECs. Although LOX-PP has been proved to have anti-tumorigenic role in various tumors^15,19^ we report for the first time the anti-angiogenic effect of LOX-PP in non-transformed endothelial cells. Thus, these findings provide therapeutic insights in vascular diseases, rheumatoid arthritis, proliferative retinopathy etc where the pathogenesis necessarily involves angiogenesis.

Collectively, these findings indicate that LOX-PP ameliorates cell proliferation, migration, cell attachment, cell cycle regulation, F-actin polymerisation, pFAK and pERK which are critical to the angiogenesis process in endothelial cells. Decrease in the vascular network formation in CAM is a clear evidence for the anti-angiogenic effect of rLOX-PP. Therefore harnessing the potential of LOX-PP can be one among the other promising strategies to inhibit angiogenesis.

## Experimental Procedures

### Ethics statement

All experiments involving the usage of human tissues were carried out according to the guidelines approved by the Institutional Review Board of the Vision Research Foundation, Chennai, India, and adhered to the guidelines of the Declaration of Helsinki. Umbilical cords were collected from donors after obtaining their informed consent.

### Cell culture

Human Umbilical Vein Endothelial Cells (HUVECs) were isolated using collagenase type-II (Sigma Aldrich, USA) digestion from human umbilical cord. The cells were cultured in 0.1% gelatin coated T-25 flask using endothelial growth medium-2 (EGM-2) supplemented with 2% FBS, VEGF, rhEGF, rhFGF-B, R3-IGF-1, gentamicin sulphate, amphotericin, hydrocortisone, heparin and ascorbic acid (Lonza, Switzerland). Cells between 2^nd^ to 6^th^ passages were used for the experiments as indicated^36^.

### Cloning of LOX-PP

The human lysyl oxidase propeptide with the signal peptide (residues 1 to 168) was amplified from HUVECs cDNA using PCR with forward primer 5′-GTTTAAACTTAAGCTTATGCGCTTCGCCTGGACC-3′ and reverse primer 5′-CCACCACACTGGATCCCTAGCCCACCATGCCGTC-3′, without signal peptide (residue 22 to 168) with forward primer 5′-GGTATCGAGGGAAGGCCTGCCCCT CC CGCCGCCGGC-3′ and reverse primer-5′-TCAGCTAATTAAGCTTCTAGCCCACC ATGCCGTC-3′. The amplified cDNA with and without signal peptide was cloned in frame into the *Bam HI* and *Hind III* sites of the mammalian expression vector pcDNA 3.1(+)/His A (Invitrogen, USA) and into the *Stu I* and *Hind III* sites of the bacterial expression vector pQE 30Xa (Qiagen, Germany) respectively using infusion cloning (Clonetech, USA). The construct was confirmed by DNA sequencing using ABI 3100 – Avant Genetic Analyzer (Applied Biosystems, USA).

### LOX-PP overexpression and purification

The recombinant LOX-PP protein was produced in M15 (pREP4) *E*.*coli* (Qiagen, Germany) using pQE 30Xa expression vector containing a 6x-Histidine tag to facilitate purification by nickel affinity chromatography. Expression of LOX-PP was induced by 1 mM of isopropyl β-D-1-thiogalactopyranoside. His - tagged protein was purified from 500 mL of *E*.*coli* overexpressed culture under denaturing conditions on a Ni - NTA agarose (Qiagen, Germany) column by binding with a buffer containing 8 M urea, 50 mM sodium dihydrogen phosphate and 300 mM sodium chloride (pH 8.0). Washing and elution was done in the above buffer containing 30 mM imidazole and 250 mM imidazole respectively. The purity of the recombinant protein was analyzed by SDS-PAGE followed by western blotting using the His tag antibody. The his tag was cleaved using Factor Xa protease (Qiagen, Germany). The identity of the protein was confirmed by 1D Nano LC - Xevo G2S QTof mass spectrometry (MS) (Waters Incorporation, USA). The spectrum was analyzed by PLGS (Protein Lynx Global Server) software version 2.5.3. (Waters Corporation, USA). For polyclonal antibody production, 300 μg of the purified LOX-PP emulsified with Freund’s complete adjuvant was administered subcutaneously (s.c.) into New Zealand female white rabbits. Booster doses of the LOX-PP antigen emulsified with Freund’s incomplete adjuvant was administered s.c. on the 21^st^, 42^nd^ and 64^th^ day and blood was collected every 14^th^ day from the first injection and booster doses. Serum from these blood samples were tested for reactivity against the purified LOX-PP protein using ELISA. The IgG fraction was purified using protein A/G agarose beads and confirmed by SDS-PAGE (Bioklone, Chennai, India). The purified recombinant LOX-PP was used to study the anti-angiogenic effect in HUVECs.

### Transient Transfection

HUVECs were transiently transfected with plasmid vectors using X-tremeGENE HP DNA transfection reagent (Roche, Canada) according to the manufacturer’s protocol. Cells were transfected with 0.75 µg of pcDNA3.1 (+)/His A + LOX-PP or empty vector (EV). After transfection the samples were incubated for 24, 48, 72 and 96 h and the expression of LOX-PP was examined at mRNA and protein levels.

### Cell viability study

MTT [3-(4,5-Dimethylthiazol-2-yl)-2,5-Diphenyltetrazolium Bromide] assay was performed to assess the toxicity of the transfection reagent in HUVECs. After incubating the cells with transfection reagent for 48 h, 20 µl of 5 mg/mL MTT reagent was added and the cells were subsequently incubated for 4 h at 37 °C until formation of formazan crystals. The formazan crystals were quantitated by dissolving in 200 µl of DMSO (Sigma Aldrich, USA) and reading the absorbance at 570 nm using a SpectroMax M2^e^ spectrophotometer (Molecular Devices, USA).

### Immunofluorescence for LOX-PP

LOX-PP overexpressed HUVECs was cultured in 24 well plates on coverslips in Endothelial growth medium (EGM-2). Subconfluent cells were then fixed in 4% paraformaldehyde for 10 min at room temperature. The cells were made permeable in 0.2% Triton-X100 and blocked in 1% BSA. After blocking, the cells were incubated with 1:100 diluted LOX-PP antibody overnight in 1% bovine serum albumin. Subsequently, the cells were incubated with appropriate secondary antibody conjugated with Alexa 488 (Invitrogen, USA) for 2 h. Omission of primary antibody was adapted as negative control. DAPI was used as the counter stain. The cells were mounted with Prolong antifade gold aqueous mounting material (Invitrogen, USA) and observed in a LSM 880 confocal laser scanning microscope (Carl Zeiss, Germany). All cell images were captured and analyzed using Zen Blue software.

### RNA Extraction and qPCR

Total RNA was extracted using the Trizol method following the manufacturer’s protocol (Sigma Aldrich, USA). Total RNA concentration was determined using Nanodrop ND-1000 spectrophotometer (Thermo Scientific, USA). cDNA was synthesized using iScript cDNA conversion kit (Biorad, USA).

Quantitative real-time PCR was performed using Applied Biosystems 7300 with SYBR Green chemistry (Applied Biosystem, USA). The LOX-PP primers were forward 5′-GGCTCACAGTACCAGCCTCA-3′, reverse 5′-AGCGGTGACTCCAGATGAGC-3′. Thermal cycler conditions for real time PCR were: initial denaturation at 95 °C for 5 min, followed by 40 cycles of denaturation at 95 °C for 15 sec, annealing at 60 °C for 20 sec and extension at 72 °C for 40 sec. Samples were run in triplicates and the data were analyzed using 2^−∆∆ct^ method with mRNA levels normalized to 18 S rRNA^37^.

### Label free relative quantification by Mass spectrometry

For mass spectrometry, 48 h of transiently transfected cells were lysed in Mammalian protein extraction reagent (M-PER) (Pierce, USA). Cell lysate was centrifuged at 10,000 rpm for 5 min. 100 µg of protein was desalted using 3 kDa cut off filter (Millipore, USA). The desalted protein was treated with 50 mM dithiothreitol (DTT) and 200 mM iodoacetamide (IAA) and subjected to insol tryptic digestion using 0.1% trypsin (Sigma Aldrich, USA) for 16 h. Dried peptides were reconstituted with 25 fmol/µl of MassPREP protein digestion standard (MPDS) and subjected to label free quantification using 1D nano liquid chromatography with Xevo G2S QTof mass spectrometry (Waters Incorporation, USA). The LC-MS^e^ raw files were analyzed using PLGS software version 2.5.3 which quantifies the relative abundance of a protein. The criteria of 2-fold increase was considered as upregulated and 2 fold decrease was considered as downregulated proteins. These proteins were annotated using the molecular integration pathway analysis by an online tool ConsensusPathBD (http://cpdb.molgen.mpg.de/).

### DNA synthesis assay

For proliferation assay, 48 h transiently transfected HUVECs with LOX-PP and EV expression plasmids were grown in 96 well plates for 24 h to reach 60% visual confluence in EGM-2, and then cultured in EBM-2 + 1% FBS for 16 h. Cells were labeled with 100 µM of BrdU and incubated for 48 h in EBM-2 with 1% FBS containing 10 ng/mL of VEGF 165 A. Experiments were terminated by fixing the cells with fix diluents. The BrdU labelled DNA conjugate was detected using the anti-BrdU-POD-Fab fragment and treated with TMB substrate to form a colour complex (Roche, Canada). This colour complex was measured spectrophotometrically using SpectraMax M2^e^ (Molecular Devices, USA) at 450 nm. The intensity of colour formation was expected to be directly proportional to cell proliferation.

### Cell cycle analysis

HUVECs transfected with LOX-PP and EV expression plasmids for 24 h were incubated with 10 ng/mL of VEGF. After the addition of VEGF, the cells were incubated for 24 h, washed twice with ice cold PBS, trypsinized and permeabilized with 30% ethanol for 30 min. The permeabilized cells were washed twice with ice cold PBS and treated with RNase (1 mg/mL) for 20 min. The cells were then incubated with propidium iodide for 30 min and analyzed by flow cytometry on a FACS calibur, (Beckman Dickinson, USA) instrument.

### Apoptosis assay

HUVECs were transfected with the LOX-PP and EV expression plasmid. After 4 h of transfection the cells were incubated with 10 ng/mL of VEGF for 48 h. The cells were then lysed and centrifuged at 20000 × g for 10 min at 4 °C to remove cellular debris. The supernatant was used in cell death ELISA kit (Roche, Canada) that measures the amount of mono and oligonucleosomes produced in the cytoplasmic fraction of lysed cells.

### Scratch assay

HUVECs (100,000 cells/well) were seeded in a 12 well plate coated with 0.1% gelatin and incubated at 37 °C with 5% CO_2_ overnight. After, 48 h post transfection, the cells were serum starved using endothelial basal medium-2 (EBM-2) containing 1% fetal bovine serum (FBS) (Gibco, USA) for 3 h. A scratch was then made manually using 200 µl tip in the confluent monolayer. The detached cells were removed by washing with 1% FBS containing EBM-2. Subsequently, fresh medium was added with 10 ng/mL of VEGF 165 A (Thermo Scientific, USA) and the cells were incubated for 16 h at 37 °C in a CO_2_ incubator. Scratch assay was also performed with rLOX-PP with 2.5 μg/mL in the presence of 10 ng/mL of VEGF. After incubation, the samples were washed and fixed in 4% paraformaldehyde, and stained with Wright Giemsa. Photographs were taken using an Axio Observer Z.1 microscope (Carl Zeiss, Germany) and the images were quantified using NIH ImageJ software^38^.

### Transwell migration assay

Transwell cell culture 8 μm inserts (Millipore, USA) were coated with 0.1% gelatin. HUVECs transiently transfected with LOX-PP and EV for 48 h were seeded with 25,000 cells/well in the upper chamber of the transwell culture insert with 1% FBS containing EBM-2. In the lower chamber, 10 ng/mL of VEGF 165 A in 1% FBS containing EBM-2 was added separately in each well. The cells were incubated for 8 h. After incubation, the transwell culture inserts were washed and the cells were fixed in 4% paraformaldehyde and stained with Wright Giemsa, following staining the cells present in the upper side of the chamber was wiped with cotton swabs and cells attached to the lower chamber were imaged using Axio Observer Z.1 microscope (Carl Zeiss, Germany). The cells were counted in the five fields manually using NIH ImageJ analysis software^39^.

### Cell attachment assay

HUVECs transiently transfected with LOX-PP and EV for 48 h were pre-treated with 10 ng/mL of VEGF 165 A for 15 min before being plated onto a fibronectin coated 24 well plates and incubated for 30 min at 37 °C. Cell attachment assay was also performed using rLOX-PP (2.5 μg/ml) in the presence of 10 ng/ml of VEGF. Attached cells were then fixed in 4% paraformaldehyde and stained with Wright Giemsa. The photographs were taken using Axio Observer Z.1 microscope (Carl Zeiss, Germany) and cell numbers quantified using NIH ImageJ software^28^.

### Actin cytoskeleton staining

HUVECs were cultured in 24 well plates on coverslip with EGM-2 medium. After 16 h, cells were serum starved for 3 h and transfected with LOX-PP plasmid. After 24 h of transfection, cells were treated with 10 ng/mL of VEGF 165 A. 18 h post treatment, cells were fixed with 4% paraformaldehyde for 10 min at room temperature. The cells were permeabilized with 0.2% Triton-X100 for 5 min and incubated with FITC-conjugated phallodin for 90 min (Abcam, United Kingdom). DAPI was used as counter stain. The cells were mounted with Prolong anti-fade gold aqueous mounting medium (Invitrogen, USA) and observed with Axio Observer Z.1 microscope (Carl Zeiss, Germany). Images were analyzed using Axio Vision Rel. 4.8 software.

### Tube formation assay

HUVECs transiently transfected for 48 h with the LOX-PP and EV expression plasmids (20,000 cells/mL) were incubated in a 1.5 mL vial containing VEGF 165 A (10 ng/mL) for 15 min at 37 °C in CO_2_ incubator. After incubation the cells were seeded on matrigel (Chemicon, USA) in 96 well plates and tube length was measured following incubation for 4 h at 37 °C in 5% CO_2_. Tube formation assay was also performed using rLOX-PP with 1 μg/ml and 2.5 μg/ml in the presence of 10 ng/ml of VEGF. The cells were fixed in 4% paraformaldehyde and images captured using an Axio Observer Z.1 microscope (Carl Zeiss, Germany). Three fields were photographed randomly in each well and the tube length was quantified using the NIH ImageJ analysis software^36^.

### Western blot

HUVEC cells were lysed in radioimmunoprecipitation assay (RIPA) lysis buffer containing 150 mM sodiumchloride, 0.1% TritonX-100, 0.5% sodium deoxycholate, 0.1% SDS (sodium dodecyl sulfate) 50 mM Tris, pH 8.0 with protease inhibitors (1 mmol/L dithiothreitol, 0.5 mmol/L phenylmethylsulfonyl fluoride, 1 mg/mLleupeptin, 10 mmol/L p-nitrophenylphosphate, 10 mmol/L h-glycerol phosphate) and phosphatase inhibitor cocktail (Cell Signaling, USA). Protein was quantified using the BCA method (Pierce, USA). 50 µg of protein was separated by SDS-PAGE and transferred to PVDF membranes (GE Healthcare, USA). Membranes were blocked in 5% Bovine serum albumin (BSA) in TBST for 1 hour and incubated with LOX-PP antibody (1:1000, Pierce, USA or in-house antibody), anti-human FAK (1:1000, Cell Signaling Technology, USA), anti-human phospho-FAK (1:1000, Cell Signaling Technology, USA), anti-human ERK, anti-human phospho-ERK, anti- His tag antibody (1:1000, Cell Signaling Technology, USA) and anti-human β – actin antibody (1:2000, Santa Cruz Biotechnology, USA) overnight at 4 °C. To detect expression of secreted recombinant LOX-PP, 1 mL of the 10 mL culture medium was subjected to immunoprecipitation with LOX-PP antibody (in-house LOX-PP antibody) and pull down was carried out using protein A/G agarose beads. Western blot analysis was performed with anti-LOX-PP antibody followed by incubation with protein A/G agarose beads. After incubation of the membranes with the primary antibodies, they were washed thrice with TBST and incubated with (1:5000), (1:10000) anti - rabbit HRP or (1:10000 and 1:20000) anti - mouse HRP conjugated secondary antibodies respectively for 2 h (Santa Cruz, USA). The membranes were developed using ECL plus reagent (GE Healthcare, USA) and the images were captured using FluorChem FC3 (ProteinSimple, USA) followed by quantification using the NIH ImageJ analysis software.

### Chick chorioallantoic membrane (CAM) assay

The chick chorioallantoic membrane assay was used to compare the *in vivo* anti-angiogenic effect of rLOX-PP based on the procedure described. Fertilized chicken eggs were kept in an incubator at 37 °C in constant humidity (65%) for 3 days. On day 4, a square hole was made on the outer shell and 0.5–1.0 ml of albumin was removed by using a 1 ml pipette to allow the detachment of the CAM membrane. Then blank filter disk containing 2.5 μg/ml of rLOX-PP, 10 ng/mL of VEGF and vehicle control were placed on top of the CAM under sterile condition. CAMs were incubated at 37 °C for 4 h. Photographs were taken using stereomicroscope (Nikon) at 0, 2 and 4 h time points. The photographs were analyzed using Angioquant software. The effect of rLOX-PP treatment was assessed by examining the alteration in the tubule length and tubule junction around the filter disks.

### Statistical analysis

All the experiments were performed independently in triplicate except proteomic analysis which was performed in duplicates. The data are expressed as mean ± SD. Student’s *t*-test was performed and one-way ANOVA analysis was done with Bonferroni correction to calculate significance. *p value* < 0.05 was considered to be statistically significant.

## Electronic supplementary material

Supplementary Dataset 1
