# Supplementary material for: Inhibition of angiogenesis in endothelial cells by Human Lysyl oxidase propeptide
Source: Sci Rep. 2018 Jul 11;8:10426. doi: 10.1038/s41598-018-28745-8 (PMC6041307; doi:10.1038/s41598-018-28745-8)
Supplement: Supplementary file 1 — Supplementary Dataset 1 [file 41598_2018_28745_MOESM1_ESM.pdf]

Supplementary Information for

Inhibition of angiogenesis in endothelial cells by Human Lysyl oxidase propeptide

Ragavachetty Nagaraj Nareshkumar[1#], Konerirajapuram Natarajan Sulochana[1] and Karunakaran Coral [1*]

[1]R.S. Mehta Jain Department of Biochemistry and Cell Biology, Vision Research Foundation, 41, College road, Chennai, India.

[#]School of Chemical and Biotechnology, SASTRA University, Thanjavur, India

Corresponding author: Karunakaran Coral

*Email id: ceeyem08@gmail.com

Supplementary Figure S1

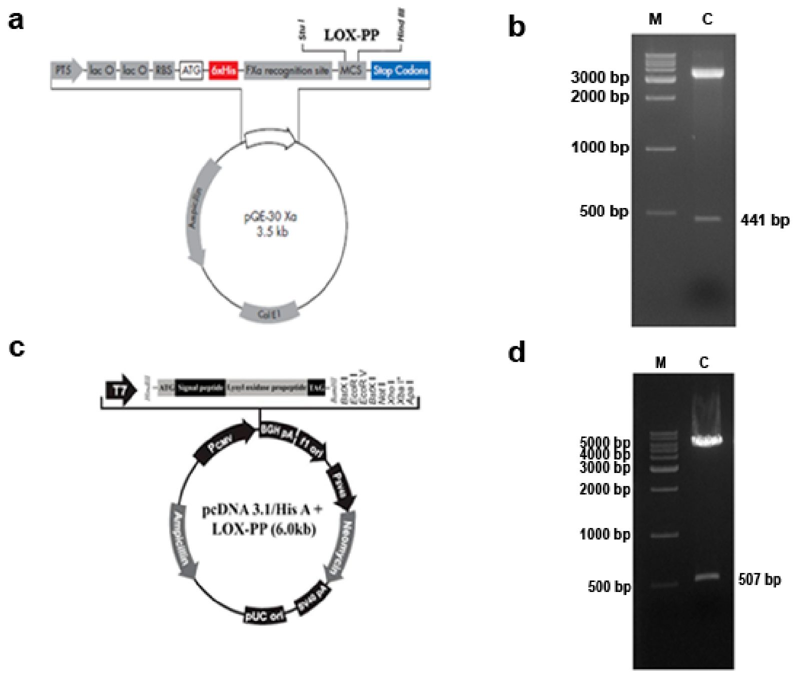

Supplementary Figure S1 Cloning of LOX-PP: (a) LOX-PP construct in pQE 30Xa. **(b)** Electrophoretogram of pQE 30Xa + LOX-PP clone digested with restriction enzymes - *Hind III* and *Stu I*. **(c)** LOX-PP construct in pcDNA3.1/His A map. **(d)** Electrophoretogram of pcDNA3.1/His A + LOX-PP clone digested with restriction enzyme - *Hind III* and *Bam HI*.

Supplementary Figure S2

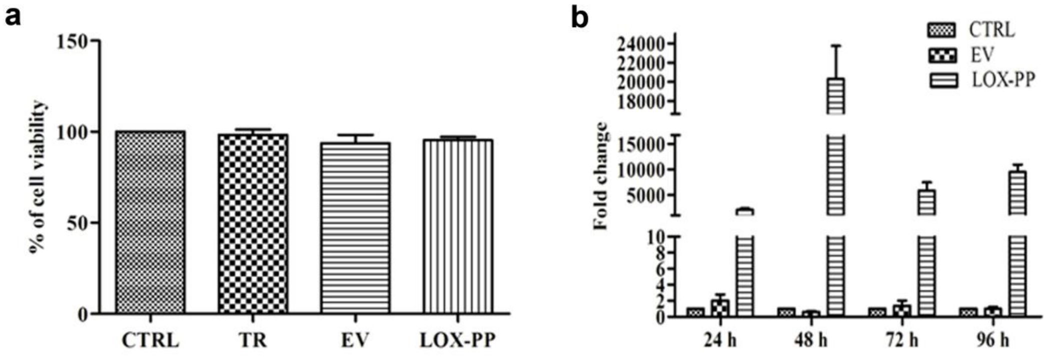

Supplementary Figure S2 Cell cytotoxicity assay: (a) LOX-PP overexpressed using transient transfection of LOX-PP construct in pcDNA 3.1(+)/HisA with Transfection Reagent (TR). The cells were also transfected with empty vector (EV), which was used as control. The cytotoxicity was quantified using MTT [3-(4,5-Dimethylthiazol-2-yl)-2,5-Diphenyltetrazolium Bromide] assay. **(b)** mRNA expression of LOX-PP upon overexpression of LOX-PP - Bar graph represents the fold change of LOX-PP in control, empty vector transfected and LOX-PP overexpressed HUVECs at 24, 48, 72 and 96 h post transfection. Values were expressed as mean ± SD, n=3.

Supplementary Figure S3

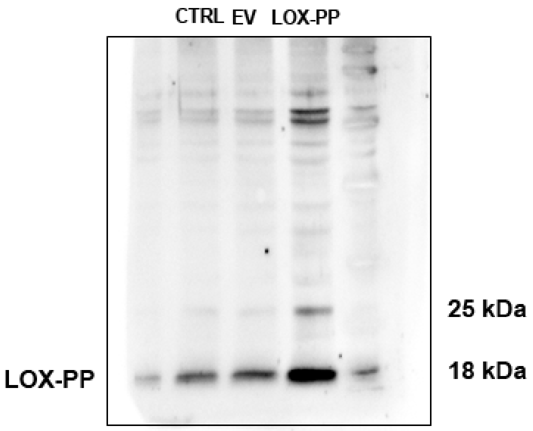

Supplementary Figure S3:

After 48 h of transfection, HUVEC cell extracts were collected and probed using LOX-PP antibody by western blot (CTRL – Control; EV- empty vector; LOX-PP –LOX-PP transfected cells). **This represents the full-length blot of figure 2b.**

Supplementary Figure S4

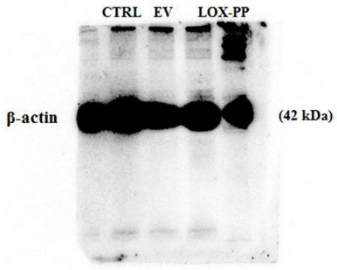

Supplementary Figure S4: After 48 h of transfection, cell extracts were collected and probed using β- actin as internal control (CTRL – Control; EV- empty vector; LOX-PP – LOX-PP transfected cells). **This represents the full-length blot of figure 2b.**

Supplementary Figure S5

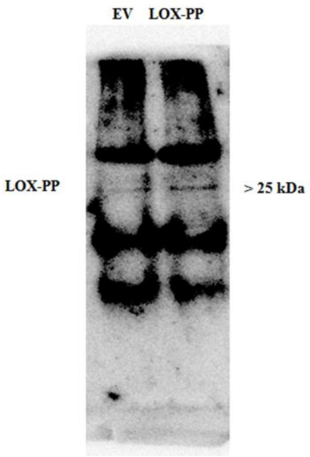

Supplementary Figure S5: Conditioned medium was collected and it was immunoprecipitated using LOX-PP antibody, immunoprecipitated samples were subjected to western blotting using antibody against LOX-PP (EV- empty vector; LOX-PP –LOX-PP transfected cells). **This represents the full-length blot of figure 2c**

Supplementary Figure S6

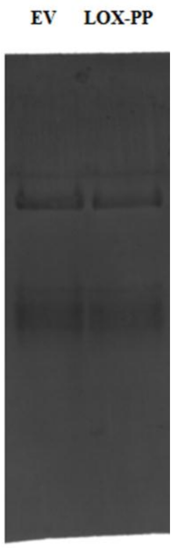

Supplementary Figure S6: Conditioned medium was collected and it was immunoprecipitated using LOX-PP antibody, immunoprecipitated samples were subjected to western blotting and coomassie staining of blot was considered as loading control. (EV-empty vector; LOX-PP –LOX-PP transfected cells). **This represents the full-length blot of figure 2c**

Supplementary Figure S7

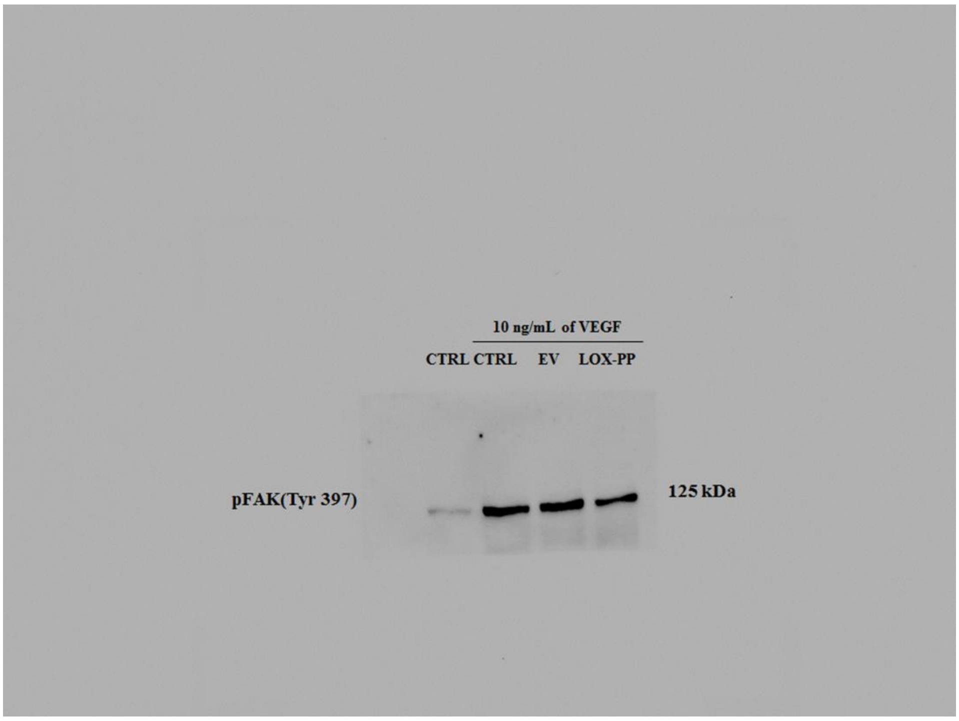

Supplementary Figure S7: HUVECs were transfected with LOX-PP and EV. After transfection, cells were treated with 10 ng/mL of VEGF for 15 min. Whole cell extract were prepared and sample were subjected to western blotting using antibodies against pFAK (Tyr397) (CTRL – Control; EV- empty vector; LOX-PP –LOX-PP transfected cells). **This represents the full-length blot of figure 9a for pFAK**

Supplementary Figure S8

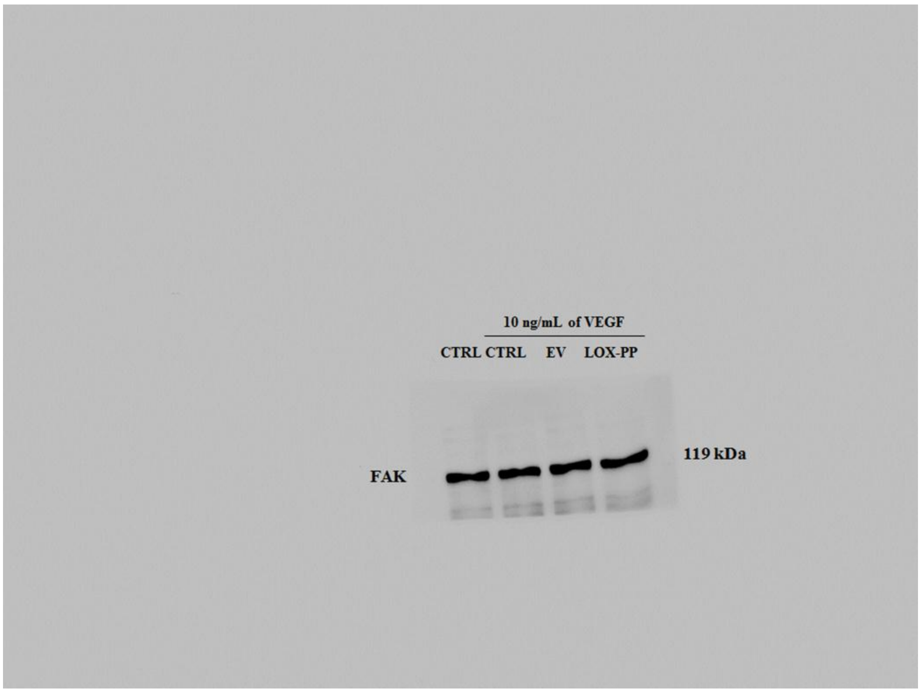

Supplementary Figure S8: HUVECs were transfected with LOX-PP and EV. After transfection, cells were treated with 10 ng/mL of VEGF for 15 min. Whole cell extract were prepared and sample were subjected to western blotting using total FAK. (CTRL – Control; EV- empty vector; LOX-PP –LOX-PP transfected cells). **This represents the full-length blot of figure 9a for Total FAK**

Supplementary Figure S9

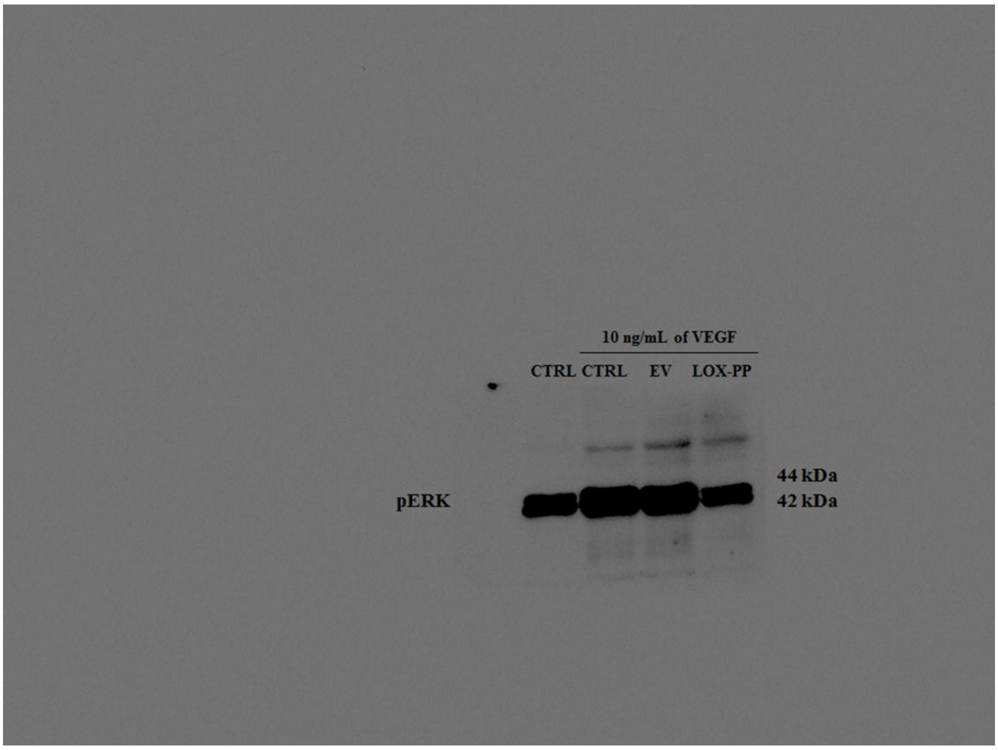

Supplementary Figure S9: HUVECs were transfected with LOX-PP and EV. After transfection, cells were treated with 10 ng/mL of VEGF for 15 min. Whole cell extract were prepared and sample were subjected to western blotting using antibodies against pERK (Thr202/Tyr204) (CTRL – Control; EV- empty vector; LOX-PP –LOX-PP transfected cells). **This represents the full-length blot of figure 9b for pERK**

Supplementary Figure S10

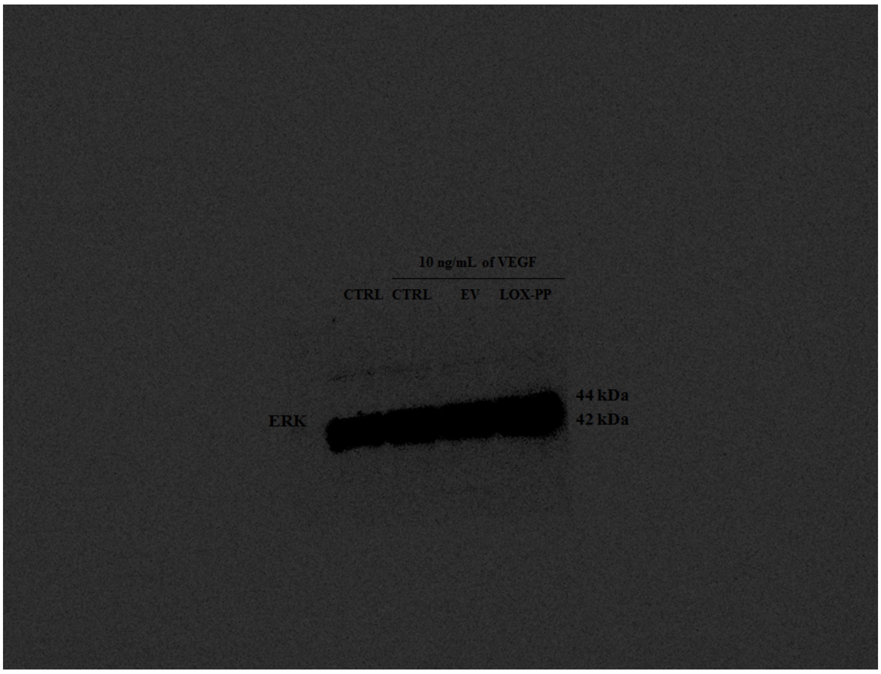

Supplementary Figure S10: HUVECs were transfected with LOX-PP and EV. After transfection, cells were treated with 10 ng/mL of VEGF for 15 min. Whole cell extract were prepared and sample were subjected to western blotting using antibodies against total ERK (CTRL – Control; EV- empty vector; LOX-PP –LOX-PP transfected cells). **This represents the full-length blot of figure 9b for Total ERK.**

Supplementary Figure S11

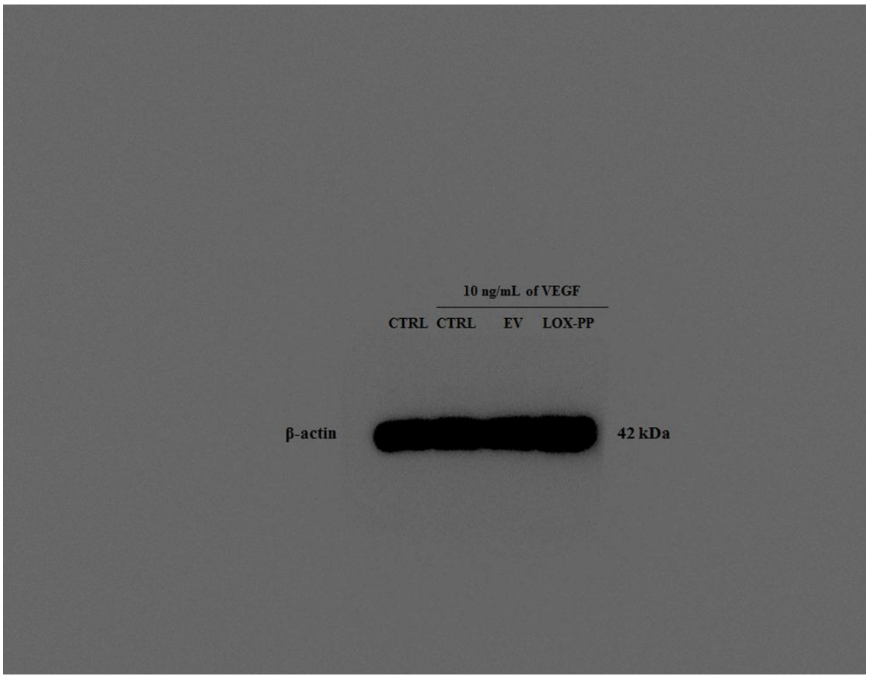

Supplementary Figure S11: HUVECs were transfected with LOX-PP and EV. After transfection, cells were treated with 10 ng/mL of VEGF for 15 min. Whole cell extract were prepared and sample were subjected to western blotting using β–actin as loading control. (CTRL – Control; EV- empty vector; LOX-PP –LOX-PP transfected cells). **This represents the full-length blots of β–actin for figure 9a and b**

Supplementary Figure S12

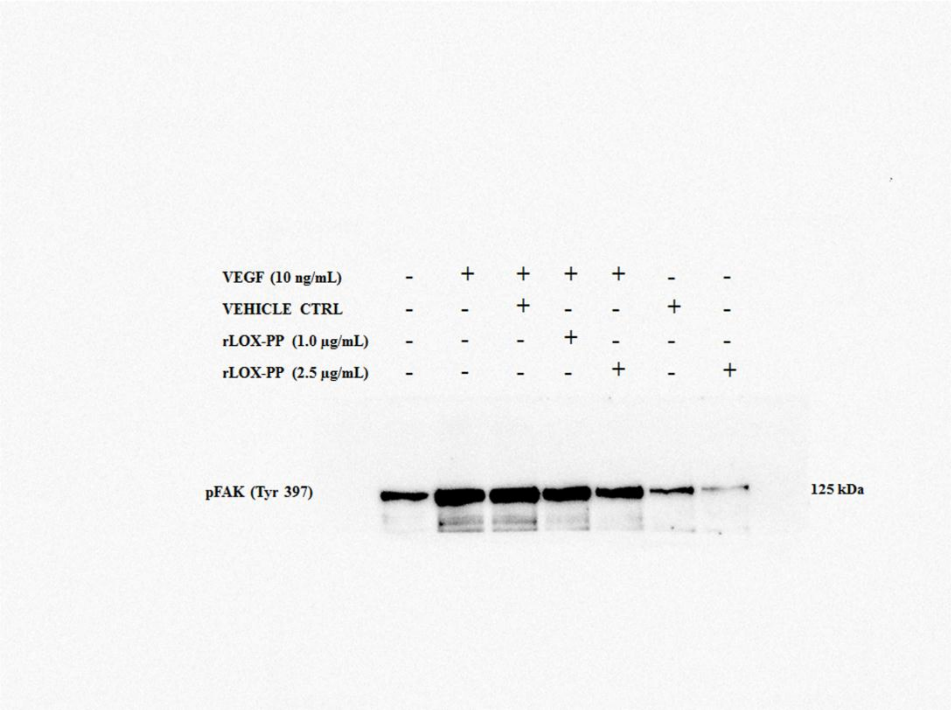

Supplementary Figure S12: HUVECs were treated with rLOX-PP and vehicle ctrl. After treatment, cells were treated with 10 ng/mL of VEGF for 15 min. Whole cell extract were prepared and sample were subjected to western blotting using antibodies against pFAK (Tyr397) (Vehicle ctrl – Vehicle control). **This represents the full-length blots of figure 10a & 10d for pFAK**

Supplementary Figure S13

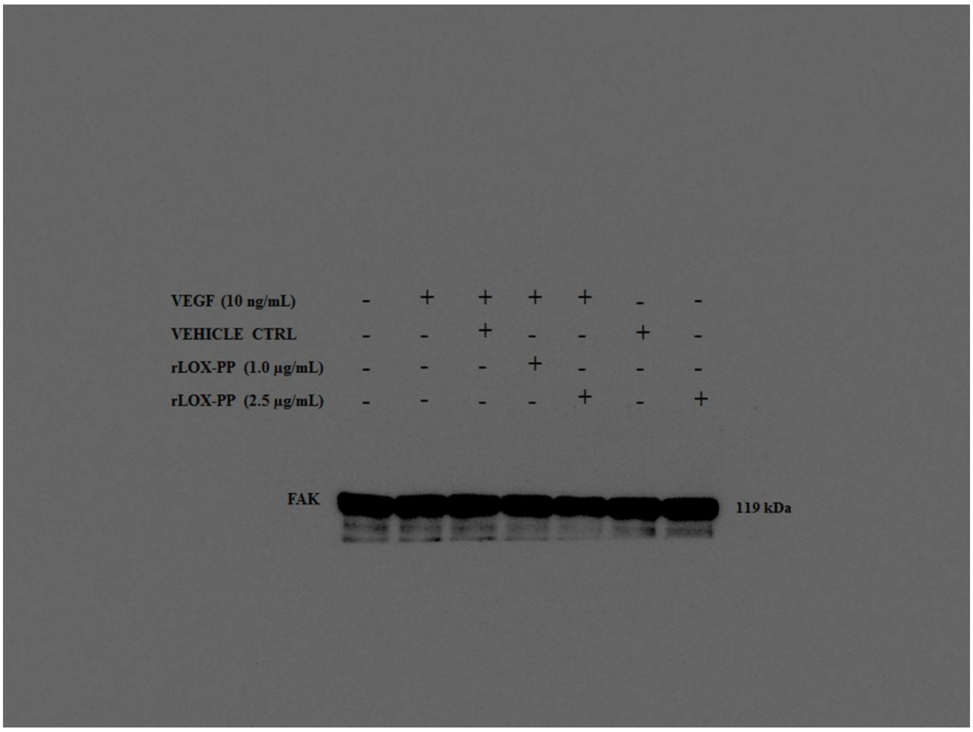

Supplementary Figure S13: HUVECs were treated with rLOX-PP and vehicle ctrl. After treatment, cells were treated with 10 ng/mL of VEGF for 15 min. Whole cell extract were prepared and sample were subjected to western blotting using antibodies against total FAK. (Vehicle ctrl – Vehicle control). **This represents the full-length blots of figure 10a & 10d for FAK**

Supplementary Figure S14

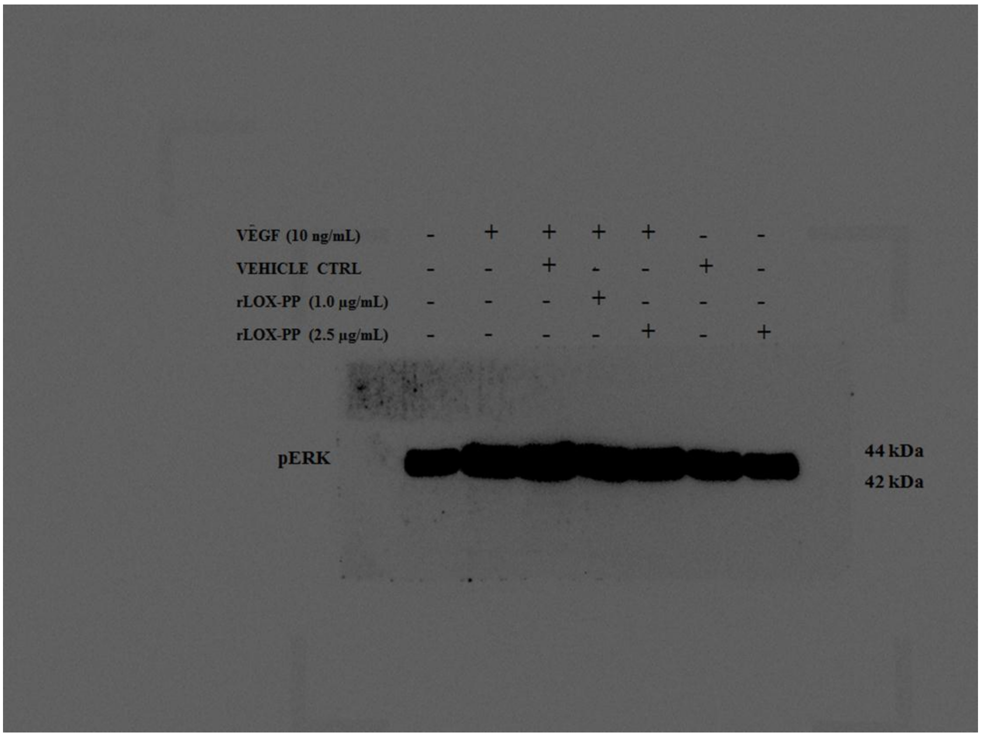

Supplementary Figure S14: HUVECs were treated with rLOX-PP and vehicle ctrl. After treatment, cells were treated with 10 ng/mL of VEGF for 15 min. Whole cell extract were prepared and sample were subjected to western blotting using antibodies against pERK (Thr202/Tyr204). (Vehicle ctrl – Vehicle control). **This represents the full-length blots of figure 10a & 10d for pERK**

Supplementary Figure S15

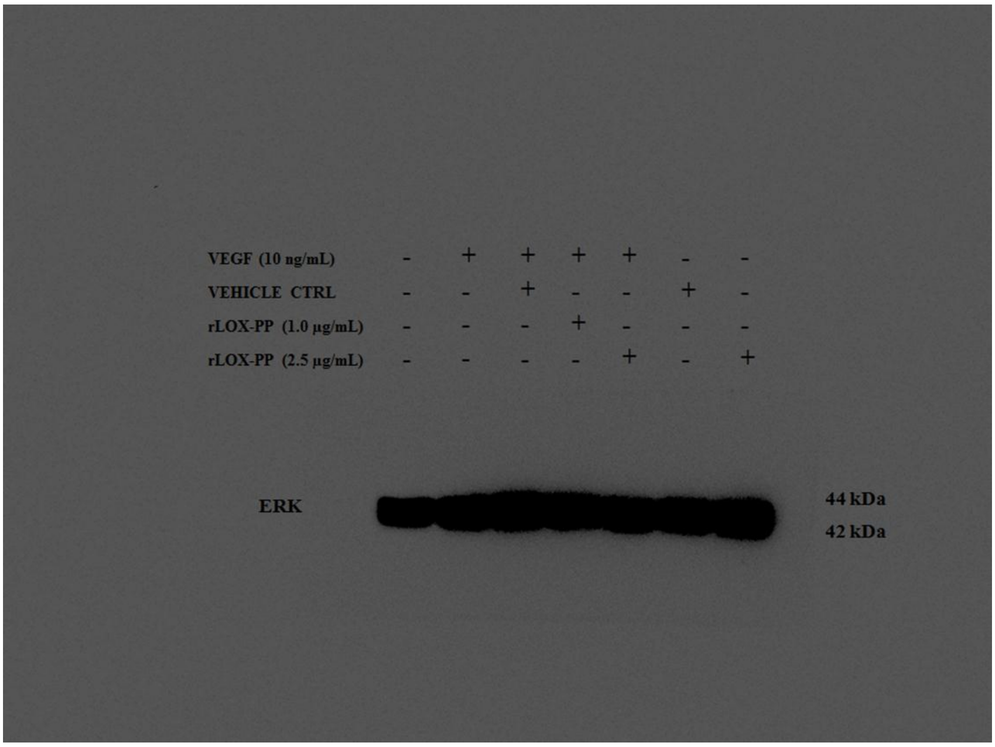

Supplementary Figure S15: HUVECs were treated with rLOX-PP and vehicle ctrl. After treatment, cells were treated with 10 ng/mL of VEGF for 15 min. Whole cell extract were prepared and sample were subjected to western blotting using antibodies against total ERK. (Vehicle ctrl – Vehicle control). **This represents the full-length blots of figure 10a & 10d for ERK.**

Supplementary Figure S16

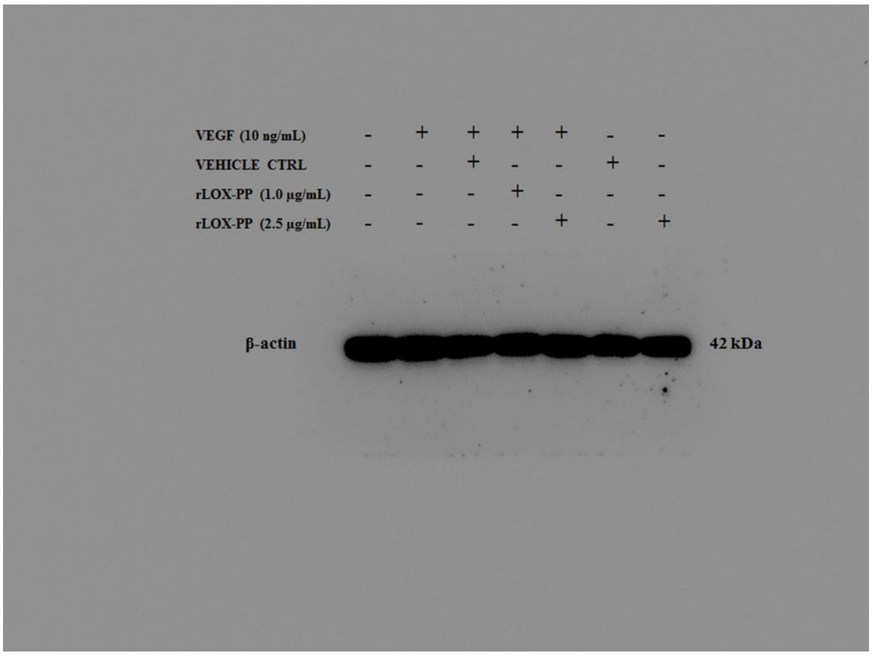

Supplementary Figure S16: HUVECs were treated with rLOX-PP and vehicle ctrl. After treatment, cells were treated with 10 ng/mL of VEGF for 15 min. Whole cell extract were prepared and sample were subjected to western blotting using β–actin which was used as loading control. (Vehicle ctrl – Vehicle control). **This represents the full-length blots of** β–actin for **figure 10a & 10d.**

Supplementary Figure S17

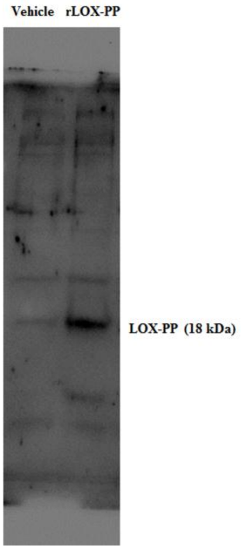

Supplementary Figure S17: Western blot of LOX-PP protein was increased upon addition of rLOX-PP in HUVECs when compared with vehicle ctrl (Vehicle ctrl – Vehicle control). **This represents the full-length blots of figure 12a for LOX-PP**

Supplementary Figure S18

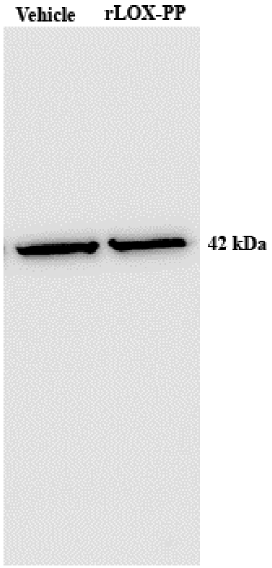

Supplementary Figure S18: Western blot of HUVECs cell lysate after addition of rLOX-PP showing β –actin used as loading control. (Vehicle ctrl – Vehicle control). **This represents the full-length blots of figure 12a for β –actin.**

Supplementary Figure S19

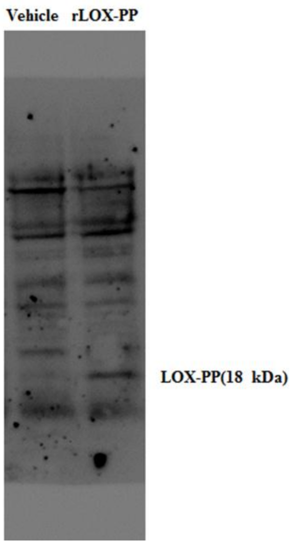

Supplementary Figure S19: Western blot for His-tag indicates rLOX-PP with His-tag which was added exogenously to HUVECs was found in the cell lysate indicating its uptake. **This represents the full-length blots of figure 12c for his-tag**

Supplementary Figure S20

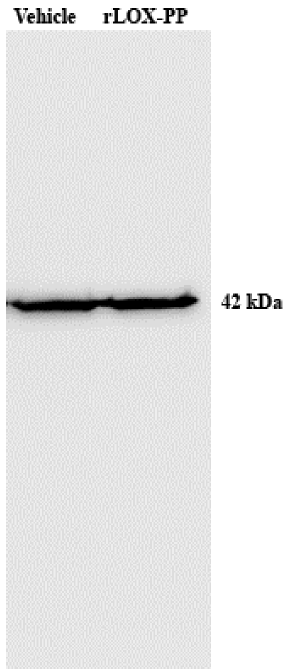

Supplementary Figure S20: Western blot of HUVECs cell lysate after addition of rLOX-PP with His-tag showing β –actin used as loading control. (Vehicle ctrl – Vehicle control). **This represents the full-length blots of figure 12c for β –actin.**

Supplementary Figure S21

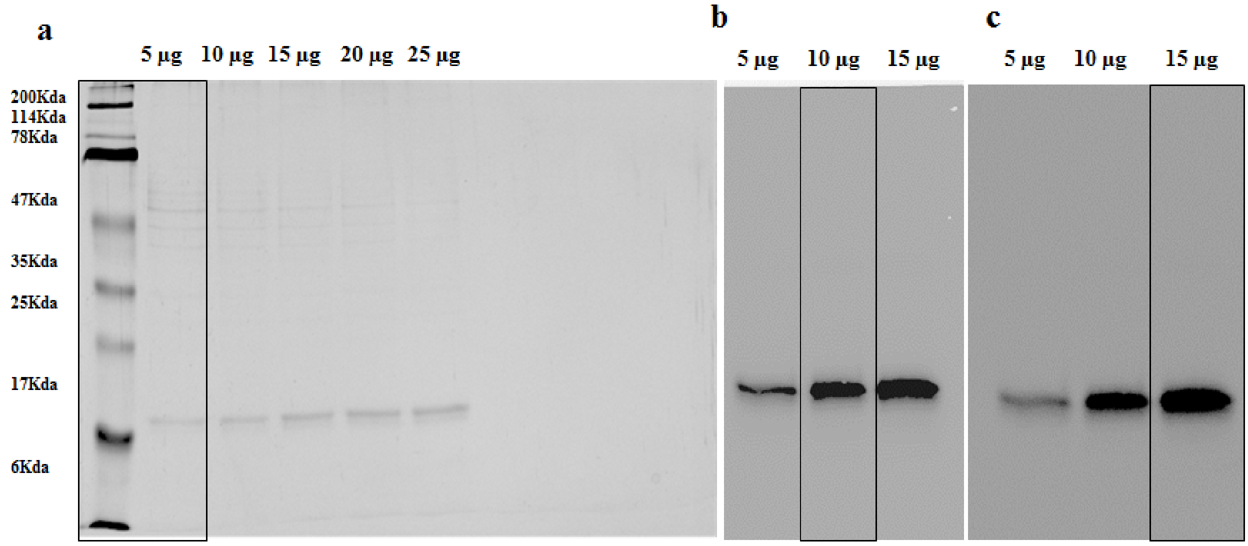

Supplementary Figure S21: Western blot for LOX-PP and His-tag in purified protein. **(a)** Different concentration of purified protein run in SDS-page and stained with coomassie stain. **(b)** Different concentration of purified protein was probed for LOX-PP. **(c)** Different concentration of purified protein was probed for His-tag. **This represents the full-length Gel/blots for figure 1b.**

Supplementary Figure S22

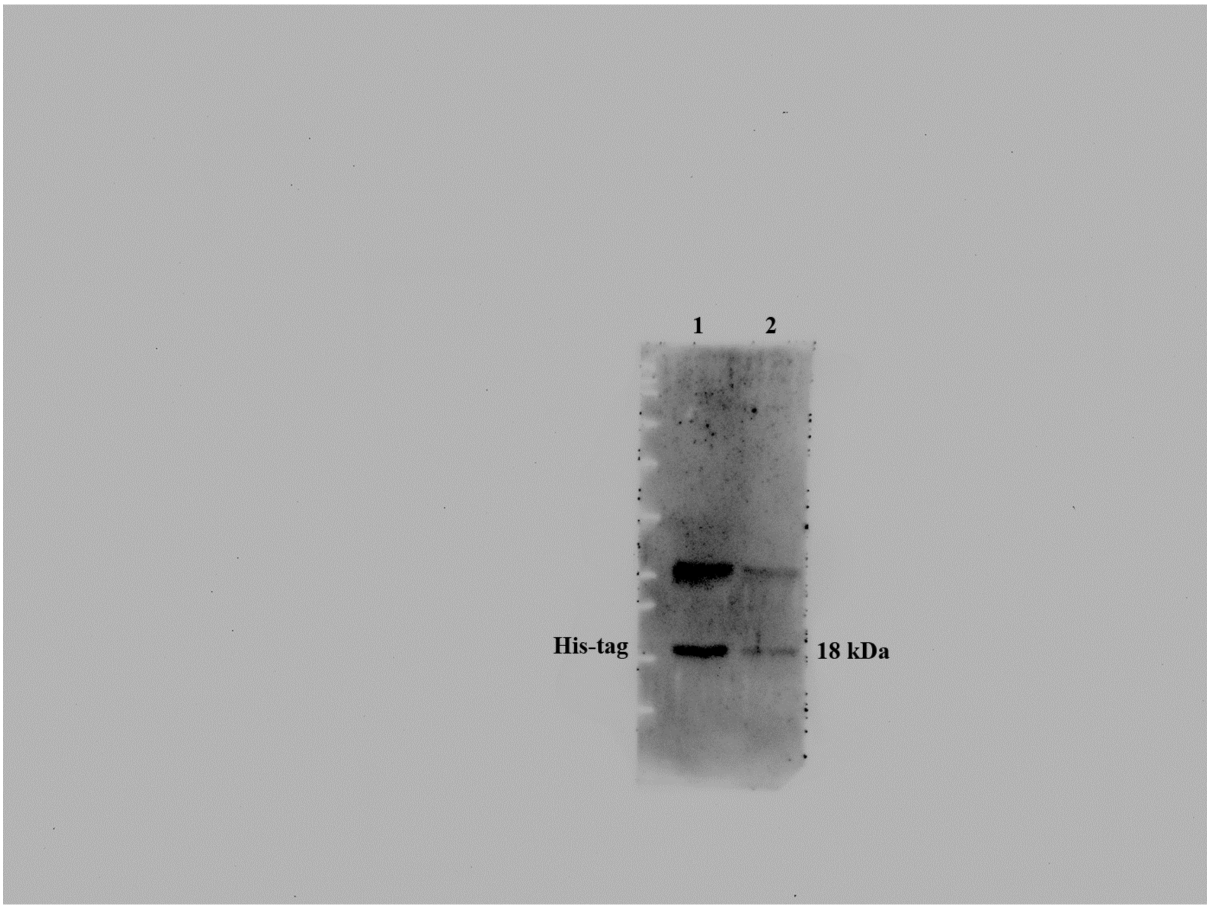

Supplementary Figure S22: Western blot for His-tag of the purified protein after His-tag cleavage using factor Xa protease (Lane-1: His-tag uncleaved, Lane-2: His-tag cleaved). **This represents the full-length blot for figure 1c.**

Supplementary Figure S23

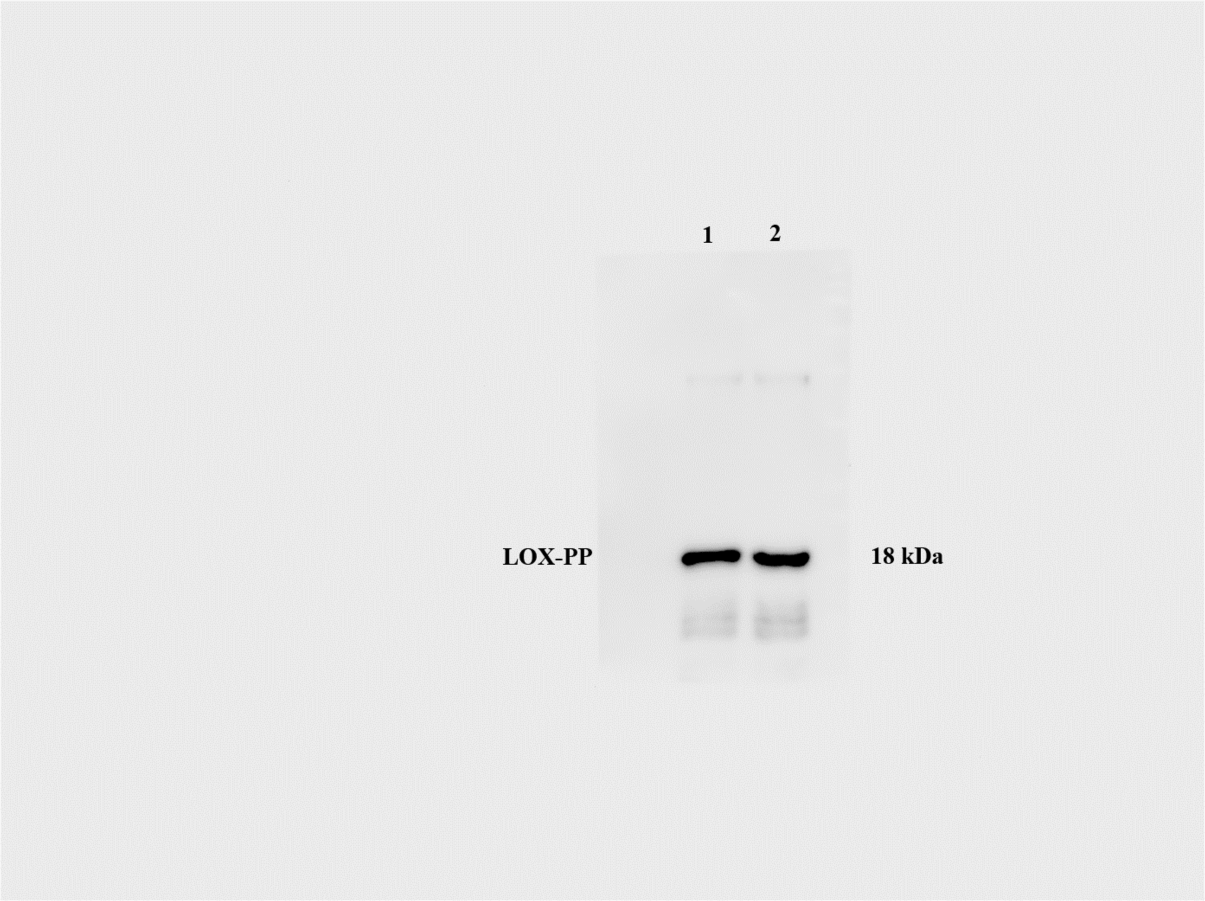

Supplementary Figure S23: Western blot for LOX-PP of the purified protein after His-tag cleavage using factor Xa protease (Lane-1: His-tag uncleaved, Lane-2: His-tag cleaved). **This represents the full-length blot for figure 1c.**
